# Directed fusion of cardiac spheroids into larger heterocellular microtissues enables investigation of cardiac action potential propagation via cardiac fibroblasts

**DOI:** 10.1371/journal.pone.0196714

**Published:** 2018-05-01

**Authors:** Tae Yun Kim, Celinda M. Kofron, Michelle E. King, Alexander R. Markes, Amenawon O. Okundaye, Zhilin Qu, Ulrike Mende, Bum-Rak Choi

**Affiliations:** 1 Cardiovascular Research Center, Cardiovascular Institute, Rhode Island Hospital and Alpert Medical School of Brown University, Providence, RI, United States of America; 2 Center for Biomedical Engineering, School of Engineering, Brown University, Providence, RI, United States of America; 3 Division of Biology and Medicine, Brown University, Providence, RI, United States of America; 4 Department of Molecular Pharmacology, Physiology and Biotechnology, Brown University, Providence, RI, United States of America; 5 Division of Cardiology, Department of Medicine, University of California, Los Angeles, CA, United States of America; Universiteit Gent, BELGIUM

## Abstract

Multicellular spheroids generated through cellular self-assembly provide cytoarchitectural complexities of native tissue including three-dimensionality, extensive cell-cell contacts, and appropriate cell-extracellular matrix interactions. They are increasingly suggested as building blocks for larger engineered tissues to achieve shapes, organization, heterogeneity, and other biomimetic complexities. Application of these tissue culture platforms is of particular importance in cardiac research as the myocardium is comprised of distinct but intermingled cell types. Here, we generated scaffold-free 3D cardiac microtissue spheroids comprised of cardiac myocytes (CMs) and/or cardiac fibroblasts (CFs) and used them as building blocks to form larger microtissues with different spatial distributions of CMs and CFs. Characterization of fusing homotypic and heterotypic spheroid pairs revealed an important influence of CFs on fusion kinetics, but most strikingly showed rapid fusion kinetics between heterotypic pairs consisting of one CF and one CM spheroid, indicating that CMs and CFs self-sort *in vitro* into the intermixed morphology found in the healthy myocardium. We then examined electrophysiological integration of fused homotypic and heterotypic microtissues by mapping action potential propagation. Heterocellular elongated microtissues which recapitulate the disproportionate CF spatial distribution seen in the infarcted myocardium showed that action potentials propagate through CF volumes albeit with significant delay. Complementary computational modeling revealed an important role of CF sodium currents and the spatial distribution of the CM-CF boundary in action potential conduction through CF volumes. Taken together, this study provides useful insights for the development of complex, heterocellular engineered 3D tissue constructs and their engraftment via tissue fusion and has implications for arrhythmogenesis in cardiac disease and repair.

## Introduction

Three-dimensional (3D) platforms bridge the gap between two-dimensional (2D) cell culture and intact tissue, since appropriate cell-cell and cell-extracellular matrix (ECM) interactions and architecture in a 3D environment are important determinants of tissue differentiation and function [1–4]. The generation of tissue culture platforms with extensive cell-cell contacts is important for a variety of biomedical engineering applications including tissue constructs for *in vivo* replacement and repair and *in vitro* models of cellular interactions with other cells, materials, and drugs. When cells are not provided with natural or synthetic surfaces or matrices to attach to, they interact with each other, aggregate, and self-organize into multicellular spheroids in a process known as self-assembly [5–7]. Spheroids have been used for disease modeling [8], regeneration [9], drug screening [10], and toxicity testing [11] using cell types from the heart [12–15], liver [16], and brain [17, 18], tumor cells [19–21], and stem-cell-derived cells [22]. Incorporation of different cell types is often needed to mimic the cellular composition of native tissue [5].

Multicellular spheroids are increasingly used as building blocks to achieve shapes, organization, heterogeneity, and other biomimetic complexities in larger engineered tissues [23, 24]. When spheroids are in close contact, they can fuse. Tissue fusion is a fundamental principle in developmental biology [25] that is very relevant to tissue engineering strategies. Little is known about how multiple cell types self-organize into distinct regions or layers as they fuse within engineered tissue or the functional behavior of cells during fusion.

Recapitulating the cytoarchitectural complexities of native tissue is important when studying the myocardium that is comprised of different cell types and shows marked differences in cellular composition and distribution depending on maturation and disease state. In the healthy adult heart, cardiomyocytes (CMs) account for 70–80% of the volume but are outnumbered by fibroblasts (CFs) and endothelial cells [26–28]. CFs are important for the architecture of the healthy heart and for reactive processes and tissue repair in disease [29, 30]. They are interspersed among CMs in the healthy myocardium [31]. In diseased hearts, CFs induce fibrosis development and their spatial distribution can be altered, depending on the nature of the insult. In pressure-overloaded hearts, CFs proliferate and produce excess ECM in between the muscle cells leading to interstitial fibrosis [32]. In contrast, when coronary blood flow is restricted in myocardial infarct (MI), CFs fill in the damaged tissue to maintain structural integrity of the heart leading to compact fibrosis [33].

Fibrotic responses affect the heart’s electrical conduction in a complex manner. Obstacles and anisotropy presented by excessive quantities of ECM proteins disturb side-to-side CM connections, reducing electrical continuity and changing conduction patterns leading to arrhythmogenesis [34–37]. Further, although CFs are non-excitable cells unable to generate action potentials (APs), important CF roles in the electrophysiology of the heart have emerged. It is important to recognize that the scar is a living tissue [38], and the influence of CFs in scar tissue on CM electrophysiology in the 3D tissue context remains understudied [39–41].

3D self-assembled models lend themselves to the investigation of electrical interaction between the two major cardiac cell types [42, 43]. We previously showed that CFs alter CM electrical characteristics in scaffold-free spheroids comprised of interspersed CMs and CFs [13] and examined the effect of CF activation commonly seen in diseased hearts on arrhythmogenicity [44]. The present study addresses how changes in CF spatial distribution seen in the infarcted myocardium affect conduction of CM electrical activity. We developed elongated 3D microtissues comprised of CMs separated by compact volumes of CFs, using individual spheroids comprised of CMs or CFs as building blocks. Since little is known about the fusion of cardiac spheroids, we first characterized fusing homotypic and heterotypic spheroid pairs. Using heterocellular elongated microtissues, we then show AP propagation through 3D volumes of CFs. Our experimental approach integrates tissue engineering and high-resolution optical mapping in a manner more controllable than in *in vivo* or whole heart experiments and is complemented by computational modeling that revealed an important role of CF ionic currents and the spatial distribution of the CM-CF boundary.

## Materials and methods

### Reagents

The following reagents were purchased from Sigma-Aldrich (St. Louis, MO): trypsin from porcine pancreas, fetal bovine serum (FBS), 100 U/ml penicillin and 100 mg/ml streptomycin, 5-bromo-2’-deoxyuridine (BrdU), sucrose, hematoxylin, and eosin. Reagents purchased from Thermo Fisher Scientific (Waltham, MA) were DMEM/F12, HEPES, trypsin/EDTA, Superfrost Plus Microscope Slides, agarose, ProLong Gold antifade reagent containing 4’,6-diamidino-2-phenylindole (DAPI), di-4-ANEPPS, Rhod2-AM, Trizol, TaqMan Reverse Transcription Reagents, TaqManGene Expression Assays, TaqMan Gene Expression master mix, and SuperSignal West Pico or Femto. The sources for all other reagents are specified below.

### Isolation, 2D culture, and adenoviral infection of neonatal rat ventricular myocytes and fibroblasts

This study was carried out in strict accordance with the recommendations in the Guide for the Care and Use of Laboratory Animals of the National Institutes of Health. The protocol was approved by the Institutional Animal Care and Use Committee of Rhode Island Hospital (protocol numbers 0057–13 and 0110–15). CMs and CFs were isolated from 2-day old (P2) Sprague-Dawley rats as previously described [44]. Briefly, isolated ventricles were digested serially with trypsin (1 mg/ml) and collagenase 2 (Worthington Biochemical Corp, Lakewood, NJ) (0.6 mg/ml) and separated into CM- and CF-enriched cell fractions by discontinuous Percoll (GE, Fairfield, CT) gradient centrifugation of the cardiac cell suspension. An automated cell counter (Countess, Thermo Fisher Scientific) was used to measure cell numbers, diameter, and viability. The frequency of cell size in 1 μm bins was recorded for 3 replicates over 7–9 isolations. The mean of the distribution from each replicate was averaged. Analysis was repeated after cells were detached (see below). CMs and CFs were plated in 2D (3x10^6^ cells/100 mm dish) with DMEM/F12 supplemented with 10% FBS, 100 U/ml penicillin, and 100 mg/ml streptomycin (referred to as “medium”). The medium for CMs was supplemented with 100 μM BrdU to inhibit the proliferation of the small fraction of CFs present (~ 5%, [44]).

After 3 days in 2D culture, cardiac cells were detached with trypsin/EDTA (3–4 min at 37°C) and counted before seeding in 3D culture (see below). Adenoviruses encoding green or red fluorescent protein (Ad-GFP or Ad-RFP) were used to visualize fusion of multicellular spheroids. To generate cardiac microtissues with Ad-infected cardiac cells, CFs or CMs were infected in serum-free DMEM/F12 for 2 hrs in suspension after trypsinization and cell counting. The cells were then collected by centrifugation and resuspended in medium 3 times before seeding in 3D culture as described below.

### Fabrication of hydrogels and generation of monoculture and coculture cardiac spheroids

**Fig 1:**
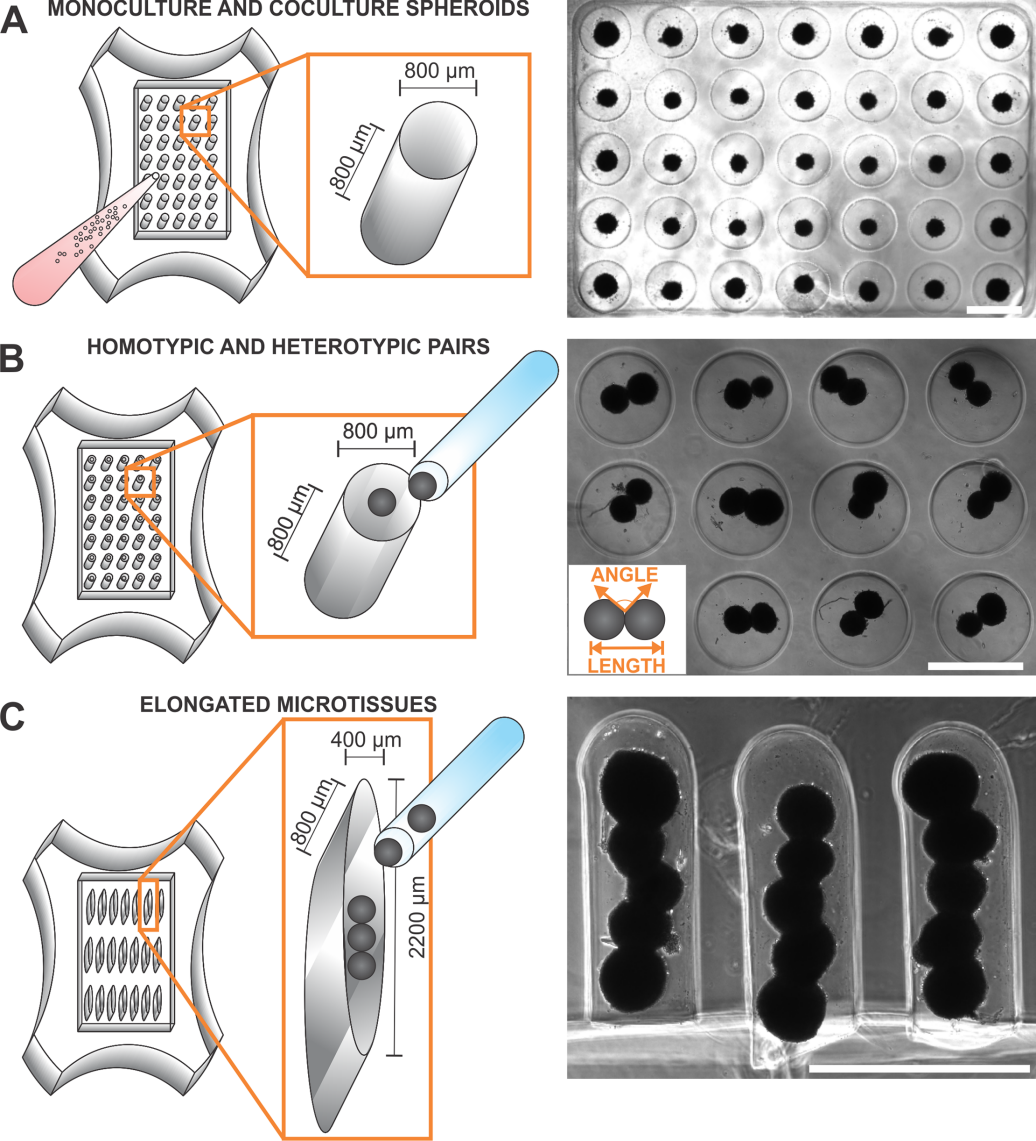
Formation of cardiac spheroids, spheroid pairs, and elongated microtissues. **(A)** 3D spheroids were generated using non-adhesive agarose gels with cylindrical recesses with hemispherical bottoms to guide self-assembly. CFs and CMs in suspension were added separately or co-seeded together to the center of the hydrogel seeding chamber to generate monoculture CM or CF spheroids or coculture CM:CF spheroids, respectively. The cells were allowed to settle into the recesses and self-assemble. **(B/C)** After 3 days, individual spheroids were transferred with a capillary tube attached to a microdispenser and micromanipulator to (**B**) cylindrical microwells containing a preformed spheroid to form homotypic (X-X where X = CM, CF, or CM:CF) or heterotypic (X-Y where X = CM and Y = CF) spheroid pairs or (**C**) to non-adhesive troughs, in which they were positioned with spheroids of variable composition in the center (CM-CM-Z-CM-CM where Z = CM or CF). Over time the spheroid building blocks fused together into longer microtissues. The phase contrast images on the right were acquired 3 days after cell seeding (**A**) and 19–20 hrs after spheroid transfer (**B/C**). Scale bars: 800 μm.

Non-adhesive agarose gels with cylindrical microwells (recesses) with hemispherical bottoms were used to guide the self-assembly of 3D microtissues (**Fig 1A**). Hydrogels were formed by pipetting sterile molten 2% wt/vol agarose into molds with 35 or 81 rounded pegs with 800-μm-diameter and 800-μm depth designed for 12- or 24-well plates, respectively (Microtissues, Inc., Providence, RI). They were allowed to solidify, released from the molds into 12- or 24-well plates, and equilibrated in medium (2 ml for 12-well or 1 ml for 24-well) overnight at 37°C in a humidified incubator with 5% CO_2_.

After 3 days in 2D culture to recover from isolation [13], CFs or CMs were detached (see above), added in monoculture or together in coculture (hereafter, CM:CF) to the hydrogel seeding chamber (0.6x10^6^ cells in 100 μL for 24-well, 1.4x10^6^ cells in 225 μL for 12-well), and allowed to settle into the recesses for 30 min. Medium was then added to each well (1 ml or 2 ml, resp.) and cells were cultured for 3 days. CM:CF coculture spheroids were plated at 1:1, 9:1, or 19:1 ratios. CM:CF(1:1) and CM:CF(9:1) were used to characterize individual spheroids and fusion of spheroid pairs. In elongated microtissue experiments, CMs were doped with an additional 5% CF (19:1 ratio, called CM) to aid in spheroid self-assembly.

### Protein and mRNA analysis in monoculture spheroids

Monoculture spheroids were generated in 12-well hydrogels for protein and mRNA analysis. After 2–3 days, they were harvested from 2–4 hydrogels for cell lysis followed by Western blot analysis or for mRNA extractions followed by quantitative PCR analysis using standard protocols for both [44]. The following rabbit monoclonal antibodies were used: α sarcomeric actinin (α-SA, 1:1000, Abcam, #ab68167, RRID:AB_11157538), GAPDH (1:1,000, Cell Signaling Technology, cat#2118, RRID:AB_561053), N-cadherin (N-Cad, 1:1000, Cell Signaling Technology, cat#13116, RRID:AB_2687616), and pan-cadherin (pan-Cad, 1:1000, Cell Signaling Technology, cat#4073, RRID:AB_2236576). In addition, a mouse monoclonal antibody recognizing vimentin (Vim, 1:500, Sigma-Aldrich, #V6630, RRID:AB_477627) was used. Stain Free technology (Bio-Rad) using a ChemiDoc MP System and Image Lab Software (Bio-Rad) was used to verify equal protein loading (10 μg per lane) and transfer to nitrocellulose membrane. FAM-labeled TaqMan MGB probes for Na^+^ channel α subunits SCN5A and SCN2A plus 18S were used to assess the fold difference in mRNA expression normalized to 18S in CF spheroids relative to CM spheroids.

### Fabrication of homotypic and heterotypic spheroid pairs

CF, CM, and CM:CF spheroids were cultured for 3 days. Two sections of a y-plate were coated with non-adhesive agarose and covered in medium as receptacles for spheroid building blocks. Hydrogels were inverted in medium to allow individual spheroids to fall out of the microwells. Individual spheroids were picked up with a capillary tube (Sutter Instrument Company) attached to a microdispenser and micromanipulator (Narishige International USA, Inc) and transferred to cylindrical recesses of a hydromold that already contained one spheroid per well and had been placed into the 3^rd^ section of the y-plate. Hydromolds containing two spheroids per microwell (**Fig 1B**) were then placed in appropriate culture plates, and the spheroid pairs were allowed to fuse over 24 hrs.

### Fabrication of elongated microtissues

CM and CF spheroids were cultured for 3 days, and transferred to y-plates as described above. Five spheroids were transferred to hydrogels with trough-shaped recesses (2200 μm length, 400 μm width, 800 μm depth; Microtissues, Inc) (**Fig 1C**). CM spheroids were positioned with spheroids of variable composition in the center (CM-CM-Z-CM-CM where Z = CF or CM). Two CM spheroids were placed on each side to ensure propagation of action potentials before they reached the center spheroid Z. This configuration also eliminated a potential influence on the membrane potential of the center spheroid by the current flow from the stimulation microelectrode. Over time the spheroid building blocks fused together into longer microtissues.

### 3D tissue sections and staining

Spheroids or spheroid pairs were fixed in PBS containing paraformaldehyde (4% PFA vol/vol, Electron Microscopy Sciences, Hatfield, PA) and sucrose (8% wt/vol), embedded, sectioned (10 μm) and postfixed (4% PFA) as previously described [44]. The cryosections were stained with hematoxylin and eosin (H&E) as previously described [44]. For immunofluorescent staining at room temperature, frozen sections were rinsed 3 times for 5 min with 1X DAKO wash buffer (Agilent, Santa Clara, CA), permeabilized with 0.2% Triton X-100 in 1X DAKO wash buffer for 5 min and rinsed 3 times for 5 min with the wash buffer. Non-specific binding was blocked with 5% goat serum for 1 hr, followed by 1 hr incubations in primary and secondary antibodies diluted in DAKO Antibody Diluent (Agilent, Santa Clara, CA). Primary antibodies were directed against α-SA (1:400, Abcam) and Vim (1:100, Sigma), and secondary antibodies were conjugated to Alexa Fluor 488 or Alexa Fluor 594 (1:200, Invitrogen). Coverslips were mounted with ProLong Gold antifade reagent containing DAPI.

### Image acquisition and processing

Phase contrast and epifluorescent images of spheroids, spheroid pairs, elongated microtissues, and tissue sections were captured with a Nikon TE2000-U and a Digital Camera (MicroVideo Instruments, Avon, MA) and acquired and analyzed with NIS Elements AR 3.2 software. Confocal images were acquired with a Nikon C1si confocal (Nikon Inc. Mellville NY) using diode lasers 402, 488 and 561. Serial optical sections were performed with EZ-C1 computer software (Nikon Inc. Mellville, NY). Z series sections were collected at 0.35 μm with a 20x PlanApo lens and a scan zoom of 2 or at 0.3 μm with a 40x PlanApo lens. Each wavelength was acquired separately by invoking frame lambda (frame sequential scanning): each laser and detector was selected for the specific wavelength, the image was collected, and all other lasers and detectors were turned off to reduce potential bleed-through when emission spectra overlap. This process was repeated for each stain and wavelength throughout a z-stack and an RGB image stack was produced. Channels were pseudo-colored and merged. Max projections of the z-stacks are presented.

### Microtissue size analysis

Stitched 4X phase contrast images of whole 24-well spheroid hydrogels were acquired and analyzed. Image thresholding and particle size analysis was employed in NIS Elements to determine the top view, cross-sectional area of individual spheroids within each mold.

### Internuclear distance analysis

20X fluorescent images of spheroids in cryosections were acquired and analyzed. Using NIS Elements, a chord was drawn across the spheroid and its length was determined. The number of nuclei intersected by the chord was counted manually. The length of the chord was divided by the number of intersected nuclei to obtain the internuclear distance.

### Timelapse and fusion analysis

4X stitched phase contrast and/or fluorescent time lapse images of fusing spheroids were acquired every hour for 19–24 hours using a humidified StageTop Incubator (Oko Lab Bold Line, Burlingame, CA) with 5% CO2 at 37°C. Length measurements were recorded manually by drawing a single line across the center of the long axis of a pair of spheroids, and angle measurements were recorded manually by selecting an anchor point on the edge of the short axis between the spheroid pair and drawing tangential lines to each of the spheroids of the pair (see insert in the image of **Fig 1B**). Angles were recorded on both sides of the fusing spheroids and averaged to represent the intersphere angle. Since our focus was not on absolute length and angle measurements, the manual approach was sufficient to effectively illustrate and compare changes in spheroid fusion over time despite potential inherent error.

### Optical mapping

The detailed description of the optical apparatus was previously published [13]. Briefly, an Olympus MVX10 microscope was used to image 1.2 x1.2 mm^2^ regions. After 7 hrs of fusion, elongated microtissues with variable composition in the center (CM-CM-Z-CM-CM where Z = CF or CM) were loaded with voltage-sensitive di-4-ANEPPS (5 μM for 10 min at 35°C), and transferred to the optical mapping apparatus. They were perfused in a temperature-regulated chamber (35°C) with Tyrode solution containing (in mmol/L) 140 NaCl, 4 KCl, 1 MgCl_2_, 5 HEPES, 0.33 NaH_2_PO_4_, 5 C_3_H_3_O_3_Na, 16 C_2_H_7_NO_3_S, 1.25 CaCl_2_, and 7.5 glucose (pH 7.4), and stimulated at one of the outer CM spheroids in the elongated microtisssue (called spheroid #1). Electrical stimulation was performed at 1 Hz with 10 ~ 50 μA strength and 2 ms stimulation duration using a glass microelectrode (Shutter Instrument, Inc.) filled with Tyrode solution. The microelectrode tip diameter was 20 ~ 30 μm and its resistance was 10 ~ 100 MΩ. Fluorescence images of membrane potentials were acquired at 979 f/s using a Photometrics Evolve +128 EMCCD camera (2x2 binning to 64x64 pixels) and a 530/30 nm bandpass filter for excitation and a 590 nm longpass filter (Semrock, Inc) for emission. Although the microtissues showed contractions (see **S1 Movie**), we were able to detect rapid action potential upstroke without a motion blocker, because we used a Tyrode solution with a moderate Ca^2+^ concentration and performed the recordings within the trough-shaped recesses that limited microtissue movement.

### Computational modeling of action potential propagation

Impulse propagation was investigated using a computational CM model and CF model. The focus of simulation was on action potential conduction and thus a generic action potential of CM is likely appropriate and insightful. We used the Luo and Rudy AP model [45] for the CMs. CFs are reported to express two types of sodium currents, Tetrodotoxin (TTX)-resistant (*I*_*Na*,*TR*_) and -sensitive (*I*_*Na*,*TS*_) [46]. *I*_*Na*,*TR*_ has rapid activation and inactivation kinetics, while *I*_*Na*,*TS*_ has higher activation V_m_ and relatively slow inactivation. Previous studies indicated that *I*_*NaTR*_ is slightly larger than *I*_*NaTS*_ in CFs [46, 47]. In this study, we used the MacCannell model of CF [48] that includes individual ionic currents so that additional sodium currents can be added and their effects on action potential propagation through CFs can be studied. We added *I*_*Na*,*TR*_ from [45] and *I*_*Na*,*TS*_ from [49] to MacCannell’s CF model, with the total CF current density being:

$$I_{CF,total}=I_{Kv,CF}+I_{K1,CF}+I_{NaK,CF}+I_{bNa,CF}+I_{Na,CF}$$

where *I*_*Kv*,*CF*_ is the time and voltage-dependent potassium current, *I*_*K1*,*CF*_ is the inward rectifying potassium current, *I*_*NaK*,*CF*_ is the CF sodium-potassium pump current, *I*_*bNa*,*CF*_ is the background sodium current, and *I*_*Na*,*CF*_ is the total voltage-dependent sodium current which is determined by the following equations:

$$I_{Na,CF}=I_{Na,TR}+I_{Na,TS}$$

$$I_{Na,TR}=g_{Na}∙m_{Na,TR}^{3}∙h_{Na,TR}∙j_{Na,TR}∙(V_{CF}-E_{Na,CF})$$

$$I_{Na,TS}=g_{Na}∙\gamma ∙\frac{F^{2}}{R∙T}∙m_{Na,TR}^{3}∙h_{Na,TS}∙[{Na}^{+}{]}_{o}∙\frac{e^{(V_{CF}-E_{Na,CF})∙F/R∙T}-1}{e^{V_{CF}∙F/R∙T}-1}{∙V}_{CF}$$

where *g*_*Na*_ is the maximum conductance of *I*_*Na*,*TR*_ and *γ* is the ratio of the maximum conductance of *I*_*Na*,*TS*_ and *I*_*Na*,*TR*_, and we set *γ* to 0.8 based on [46, 47].

**Fig 2:**
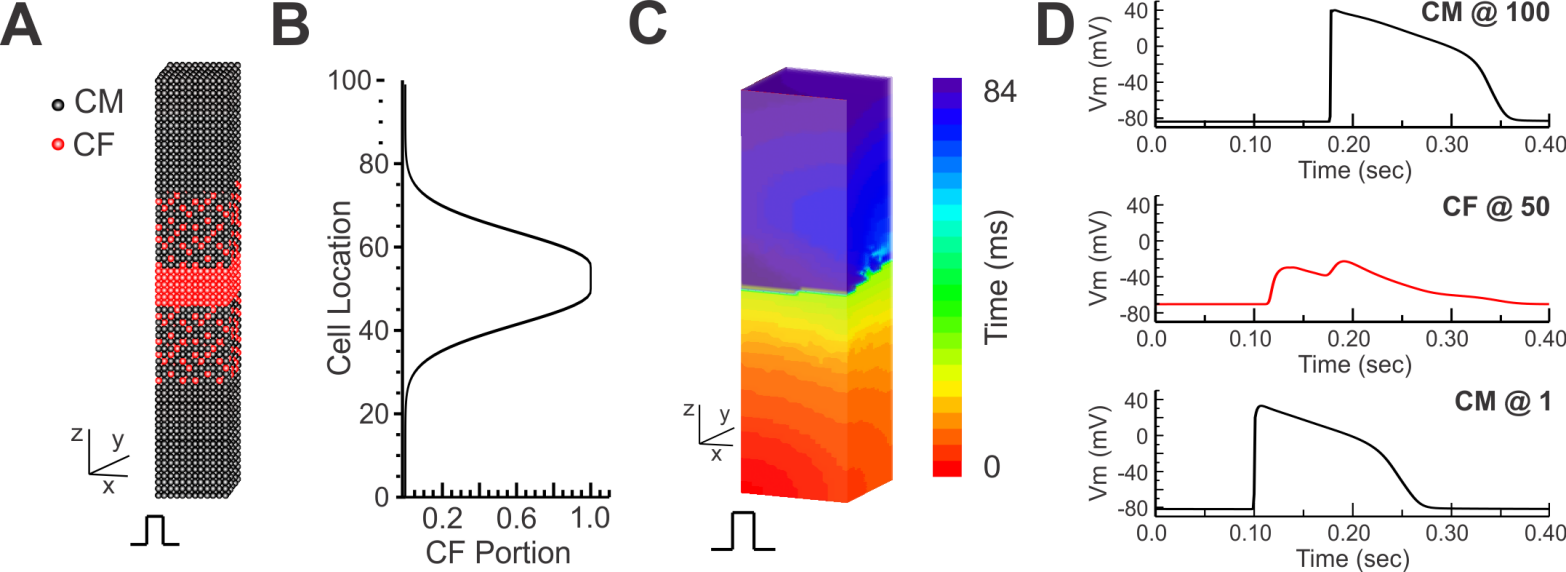
Schematics of 3D computer modeling. **(A/B)** Schematics of 3D box modeling. One spheroid was modeled with a cube composed of 20×20×20 cells. Five spheroids were connected in series to have 20×20×100 cells. The middle spheroid was composed of CFs only, the boundary was composed of mixed CM and CF cells, and the remainder of the cells were CMs. The density profile is shown in panel B. The CF distribution is followed with Gaussian distribution in both border regions. **(C)** Example of an AP propagation map in the 3D box model. The simulated tissue was stimulated from the bottom layer as indicated. **(D)** Representative V_m_ traces from a CM at cell location 1 (CM @1), a CF at location 50 (CF @50), and a CM at location 100 (CM @100) along the propagation line.

The single spheroid typically has 20–25 cells across its diameter and in our computer model, each spheroid was composed of 20x20x20 cells. The elongated tissue with 5 spheroids was constructed as a 3D rectangular prism of 20×20×100 cells (**Fig 2A**). To replicate the migration of CFs into CM spheroids in the boundary between CM and CF spheroids, the number of CFs was increased gradually in each layer of 20x20 = 400 cells with Gaussian function (# of CFs at location z: $nCF(z)=400\times \frac{1}{\sigma \sqrt{2\pi }}e^{-\frac{1}{2}{\left(\frac{z-z_{0}}{\sigma }\right)}^{2}}$), and the CFs were randomly distributed in the layer (**Fig 2B**). Each cell was electrotonically connected to up to 6 neighboring cells in the x, y, and z directions through gap junctions (monodomain model). The membrane potential (V_m_) of a CM or a CF is described by the following differential equation:

$$C_{i}\frac{dV_{m,i}}{dt}=-I_{ion,i}+\sum_{k=1}^{n}g_{gap,i-k}(V_{m,k}-V_{m,i})$$

where *C*_i_ is the membrane capacitance of the *i*th CM or CF, *I*_ion,i_ is the corresponding ionic current, n is the number of coupled cells to the *i*th CM or CF, *g*_*gap*,*i-k*_ is the gap junction conductance between the *i*th cell whose membrane potential is *V*_*m*_,_i_ and *k*^th^ connected cell whose membrane potential is *V*_*m*,*k*_. The g_*gap*,*CM-CM*_ was set to 200 nS, and the g_*gap*,*CM-CF*_ and g_*gap*,*CF-CF*_ were set to 10 nS (5% of g_gap,CM-CM_) and 20 nS, respectively. The current stimulation was applied to the CMs in one side of the 3D box for induction of AP propagation. The capacitances of CM and CF were set to *C*_*CM*_ *= 63 pF* and *C*_*CF*_ *= 6*.*3 pF*, respectively.

Numerical calculation of these differential equations was performed using a forward Euler method and the Rush-Larsen method with *Δt = 1 ~ 5 μs* time step. Simulations were carried out on a multi-core graphic processing unit (Geforce GTX Titan, NVidia) with double precision accuracy. The routines were written in a custom-developed software using Interactive Data Language (IDL) programing environment (Harris GeoSpatial Solutions, Inc.) and CUDA toolkit (Nvidia). An example of a space-time plot of V_m_ showing AP propagation is shown in **Fig 2C**, and AP traces from three different regions are shown in **Fig 2D**. The simulation code is available on-line (**S1 Appendix**).

### Statistical analyses

Values are presented as means±SD. N is reported as number of cells/spheroids/pairs, number of replicates, and number of repeated experiments where appropriate and as indicated. Statistical analyses were performed using one-way ANOVA with Bonferroni post-hoc tests, Student’s two-tailed paired and unpaired t-test, or Fisher’s exact test as appropriate.

## Results

### Characterization of spheroid building blocks

**Fig 3:**
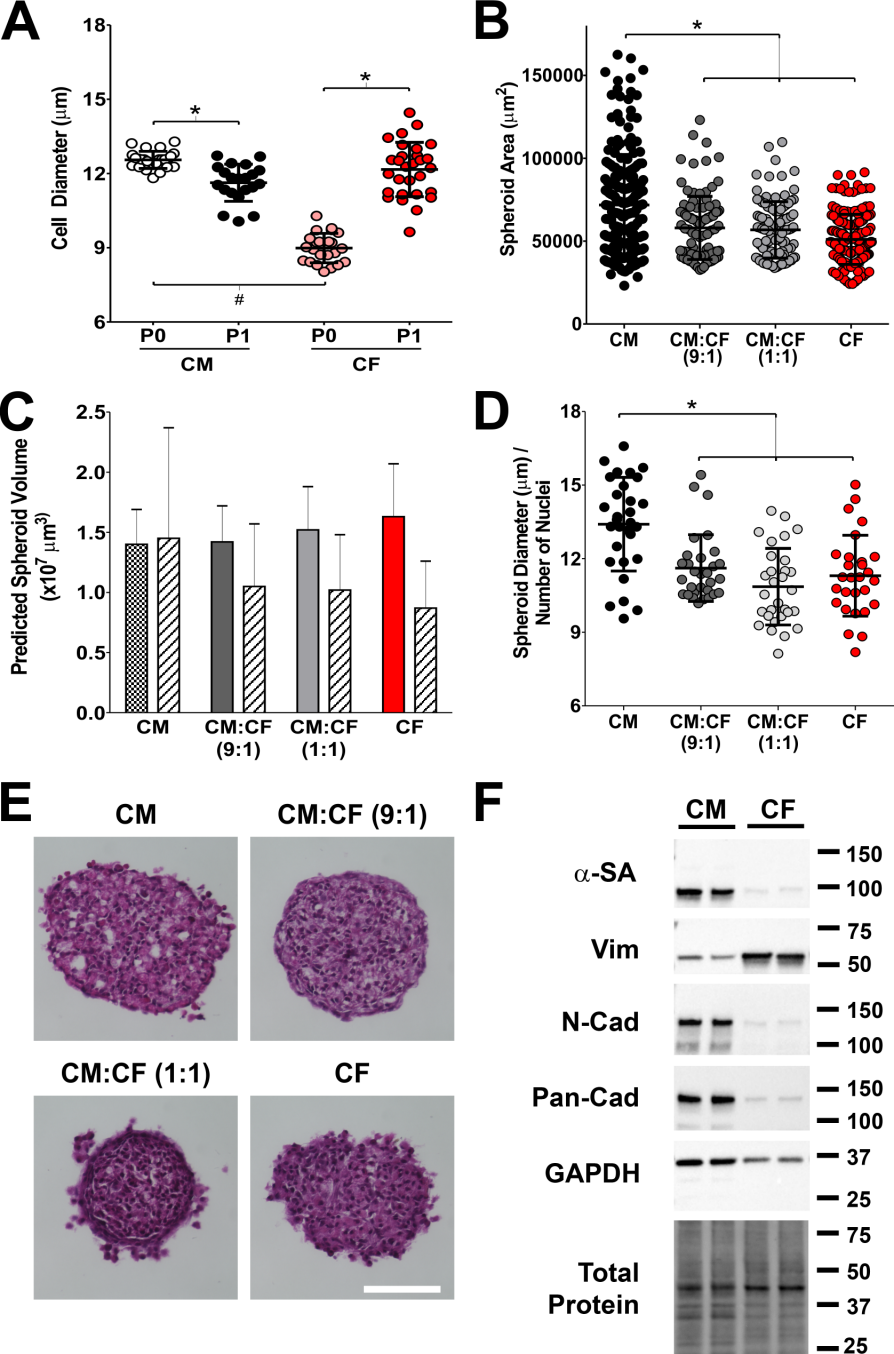
CM and CF cell size changes with passage and 3D culture. **(A)** Cell diameter of CMs and CFs that were freshly isolated (P0) or precultured for 3 days (P1), recorded with a Countess Automated Cell Counter (n = 7–9 isolations). Mean ± SD. *,^#^ p<0.05 (ANOVA). **(B)** P1 CFs or CMs were plated in 24-well hydromolds with 35 recesses at 0.6x10^6^ cells/mold. After 3 days in 3D culture, spheroids were imaged and analyzed (N = 178–209 spheroids) for circular cross-sectional area. **(C)** Assuming spherical cell shape, spheroid volume was calculated from the cell diameters in A (solid bars) or spheroid areas in B (hatched bars). **(D)** Internuclear distance in DAPI-stained cryosections of spheroids of indicated composition (generated as in B), expressed as chord length across the spheroid/number of intersected nuclei (N = 28–30 spheroids). **(E)** H&E stained cryosections of monoculture and coculture spheroids of indicated compositions. Scale bar: 100 μm. **(F)** Representative Western blots of spheroid lysates (10 μg protein/lane) obtained after 2 days in 3D culture and probed with indicated antibodies. α-SA– α sarcomeric actinin; Vim–vimentin; N-Cad–N-cadherin; Pan-Cad–pan-cadherin; GAPDH–glyceraldehyde 3-phosphate dehydrogenase. Total protein visualized by Stain Free Technology (Bio-Rad) was used as loading control.

Our goal was to generate elongated cardiac microtissues with CMs separated by compact volumes of CFs as seen in the ventricular myocardium post infarct. We used directed fusion of individual scaffold-free spheroids comprised of CMs and CFs as building blocks to achieve the goal. First, we characterized the cellular inputs and aggregated spheroid building blocks (**Fig 3**). Freshly isolated CMs (Passage 0, P0) were significantly larger in diameter than freshly isolated CFs (**Fig 3A**). After 3 days in 2D culture and detachment from the culture plate (Passage 1, P1), CF size significantly increased by 36% to 12.2 ± 1.1 μm, and CM size significantly decreased by 8% to 11.6 ± 0.8 μm, leading to comparable cell diameters of P1 CMs and CFs that were used to generate monoculture and coculture spheroids. Despite comparable input cell size, the cross sectional area of self-assembled spheroids was significantly larger for CM than for CM:CF(9:1), CM:CF(1:1) and CF spheroids (**Fig 3B**), indicating that the addition of just 10% CFs to CM monocultures [CM:CF(9:1)] significantly reduces spheroid size.

Assuming spherical cellular morphology, the input size of CMs (**Fig 3A**) and the measured area of CM spheroids (**Fig 3B**) predict similar spheroid volumes (**Fig 3C**). In contrast, with increasing CF fractions incorporated into the spheroids, the predicted spheroid volume based on the measured spheroid area was progressively smaller compared to the prediction based on input cell size (**Fig 3C**). As this could suggest that CFs pack together more tightly than CMs in spheroids, we cross-sectioned spheroids to count the number of nuclei across the spheroid diameter. **Fig 3D** shows that the internuclear distance was greater in CM than that in CF-containing spheroids. Although not quantitative, the gross morphology of H&E-stained cross sections also suggested that CM spheroids were more loosely packed (**Fig 3E**).

We next examined protein expression of known markers for CMs (α-SA) and CFs (Vim), and cell adhesion proteins (N-cadherin, pan-cadherin) known to be involved in self-assembly and cell sorting (**Fig 3F)**. As expected, α-SA and Vim were primarily detected in CM and CF spheroids, respectively. N-cadherin and pan-cadherin were much more prevalent in CM spheroids than in CF spheroids.

### Fusion of homotypic and heterotypic cardiac spheroids

**Fig 4:**
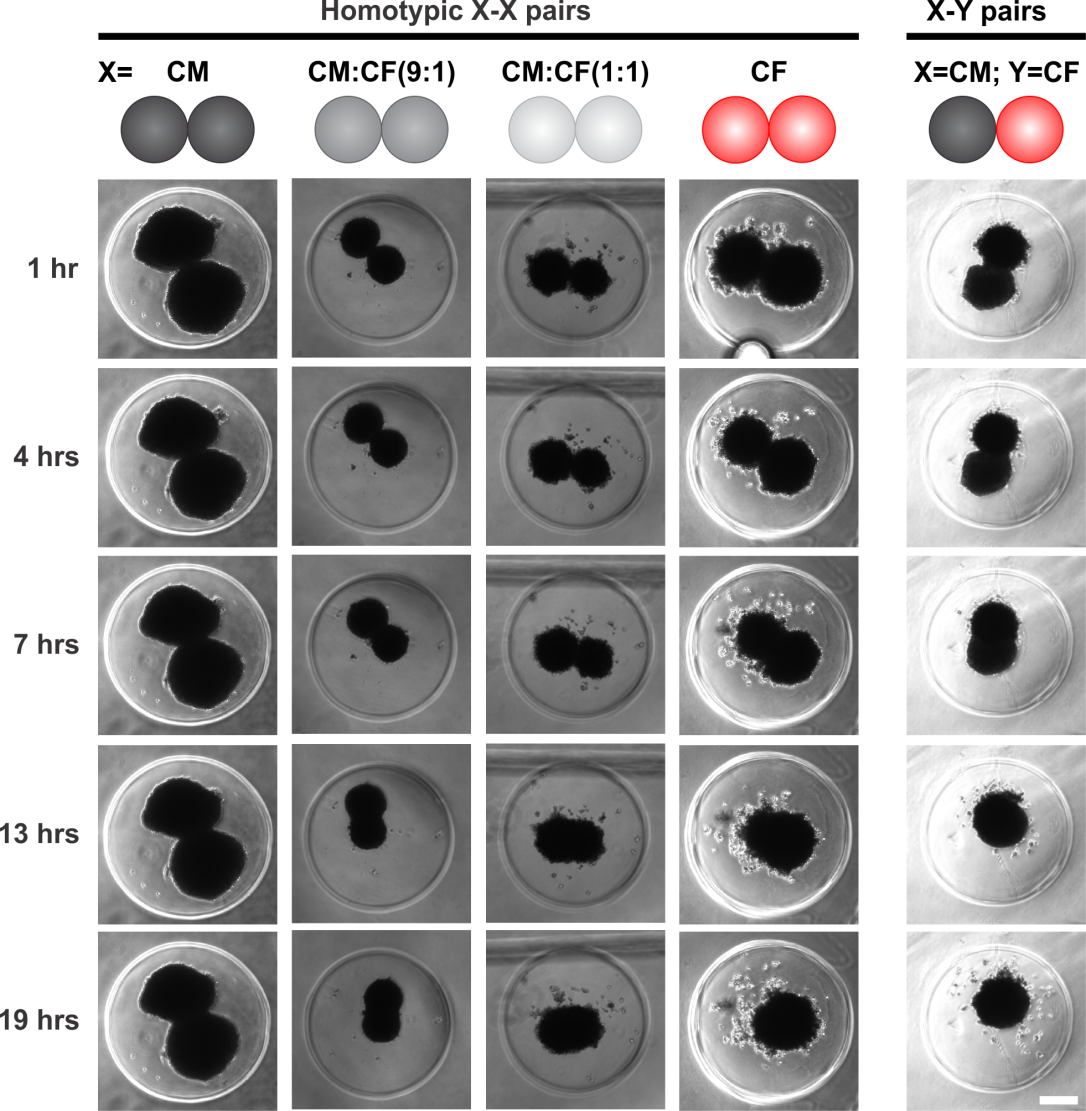
Time course of self-directed assembly of homo- and heterotypic pairs of cardiac spheroids. After 3 days in 3D culture as individual spheroids, CM, CM:CF (at 9:1 and 1:1 ratios), and CF microtissues were replated in pairs and allowed to fuse. Representative phase contrast images acquired over 19 hrs post assembly of homotypic (X-X) pairs, in which X are either CM, CM:CF(9:1), CM:CF(1:1), or CF spheroids, and heterotypic (X-Y) pairs comprised of a CM and a CF spheroid. Scale bar: 200 μm.

**Fig 5:**
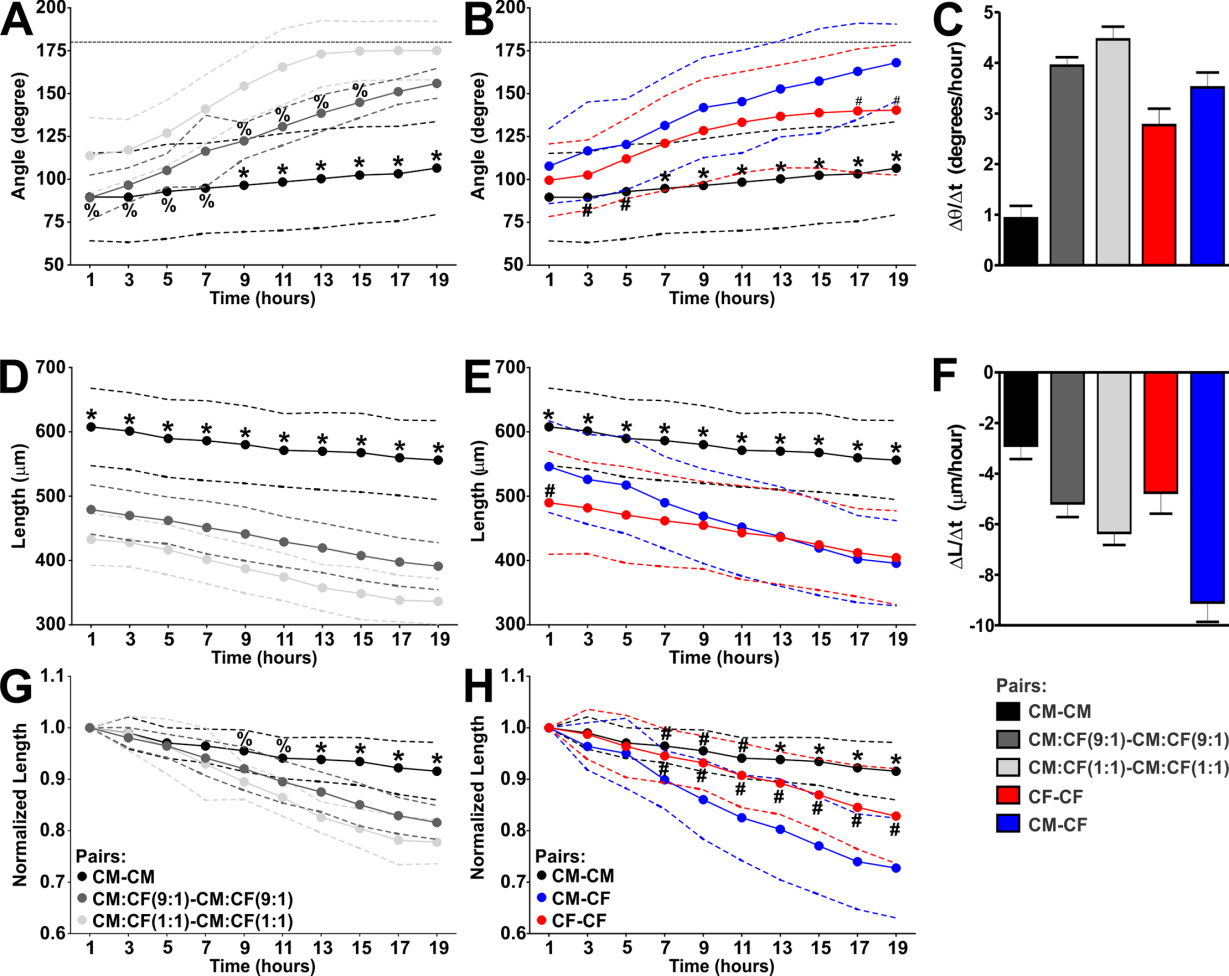
Time course of changes in intersphere angle and length of homotypic and heterotypic spheroid pairs. Intersphere angle **(A/B)** and long axis length **(D/E**) of indicated spheroid pairs that were generated as for Fig 4. Length is also presented normalized to pair length at 1 hr after replating **(G/H)**. Homotypic (X-X) pairs: CM pairs (black, n = 51–52), CM:CF(9:1) pairs (dark grey, n = 21), CM:CF(1:1) pairs (light grey, n = 26), CF pairs (red, n = 31); heterotypic (X-Y) pairs: CM-CF pairs (blue, n = 37). Mean (solid line) ± SD (dashed lines). The original values behind the means and SD are provided in **S2 Appendix**. Linear regression analysis was carried out on each data set, and the regression coefficients are presented in bar graphs **(C, F)**. * p<0.05 vs. ALL, ^%^ p<0.05 vs. CM:CF(1:1) pairs, ^#^ p<0.05 vs. CM-CF pairs.

Placement of individual spheroids in close proximity in non-adhesive trough hydromolds allowed for fusion and generation of elongated microtissues (**Fig 1B and 1C**). To inform the time course of generation of larger microtissues, spheroid pair fusion was studied first by acquiring images over 19 hrs (**Fig 4, S2 Movie**) and by determining the angle between spheroids which approaches 180° as they fuse [50, 51] and the long axis length of the fusing spheroids (**Fig 5**). The angle between homotypic CM pairs only increased from 89.7±25.5° to 106.5±27.1° over 19 hrs (**Fig 5A**, also depicted in **Fig 5B** as reference) at a rate of 0.95±0.20°/hr (**Fig 5C**). Incorporation of 10% and 50% CFs into CM spheroids prior to fusion markedly facilitated spheroid fusion: while the initial angle between CM:CF(9:1) spheroid pairs was comparable to CM spheroids **(Fig 5A**), the rate at which their intersphere angle increased was accelerated 4.0 fold (**Fig 5C**), and this angle was significantly larger than for CM pairs as of 9 hrs (**Fig 5A).** The initial angle of CM:CF(1:1) pairs was already more obtuse than CM and CM:CF(9:1) pairs (**Fig 5A**), and the intersphere angle rate increased 4.3 fold (**Fig 5C**). In contrast, the rate of change in CF spheroid pairs was 2.6 fold faster than CM pairs (**Fig 5B and 5C**). Heterotypic spheroid pair (CM-CF) fusion analysis revealed that while their initial intersphere angle was not significantly different from their homotypic counterparts (**Fig 5B**), the rate of change was 3.6 fold faster than CM pairs and 1.4 fold faster than CF pairs (**Fig 5C**). After 19 hrs, CM-CF pairs had a more obtuse intersphere angle (168.1±22.5°) than CF pairs (140.4±37.8°), and both were larger than CM pairs (106.5±27.1°) (**Fig 5B**).

We also analyzed the change in length of spheroid pairs as they fused (**Fig 5D–5H**), which was reflected in a decrease of their combined length over time. Consistent with the larger size of single CM spheroids (**Fig 3B**), CM pairs were longest in initial length by 11.3%-40.4% compared to all other conditions (**Fig 5D and 5E**). We therefore normalized length measurements to the initial length for each condition (**Fig 5G and 5H**). Consistent with the very modest changes in intersphere angle of CM pairs (see above), they showed the slowest change in length over time with a rate of -2.80 ± 0.46 μm/hr (**Fig 5F**). The normalized length of CM pairs after 19 hrs was still 91.6±5.6% of the initial length (**Fig 5G and 5H**). Incorporation of CFs into spheroids [CM:CF(9:1) and CM:CF(1:1)] prior to pair assembly accelerated fusion 1.8 fold and 2.1 fold, respectively, compared to CM pairs (**Fig 5F**). The normalized length of CM:CF(9:1) and CM:CF(1:1) pairs was 81.6±3.3% and 77.7±4.2% of their initial length after 19 hrs, respectively (**Fig 5G**). Homotypic CF pairs fused 1.7 fold faster than CM pairs but slower than those with mixed cell composition (**Fig 5F**). The fastest shortening of microtissue length was seen in heterotypic CM-CF pairs (1.4–3.1 times faster than all other conditions at a rate of -8.73±0.65 μm/hr, **Fig 5F**). The length of fused CM-CF microtissues after 19 hrs was 72.7±9.7% of their initial length (**Fig 5H**). Taken together, homotypic CF pairs fuse faster than homotypic CM pairs, but co-culture of CMs with just 10% CFs suffices to markedly increases the rate of CM fusion. Most striking was the rapid kinetics of fusion between heterotypic CM-CF spheroid pairs.

### Cell sorting of myocytes and fibroblasts in fused tissue pairs

**Fig 6:**
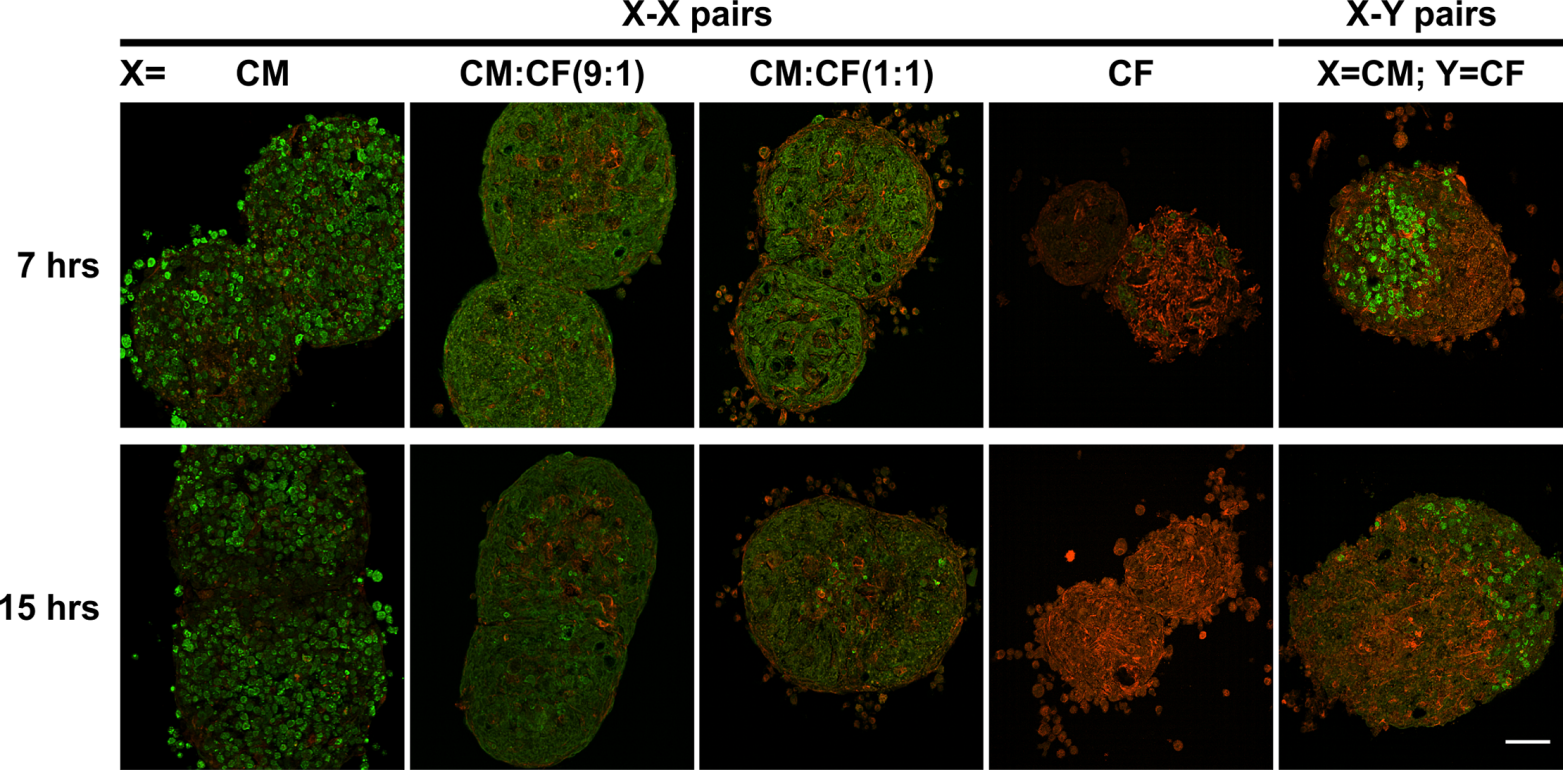
CM and CF distribution in homotypic and heterotypic spheroid pairs. Cryosections of homotypic (X-X) and heterotypic (X-Y) pairs of spheroids of indicated cellular compositions were fluorescently double-stained with antibodies recognizing α-sarcomeric actinin (α-SA) and vimentin (Vim) to visualize the CMs (green) and CFs (red), respectively. Merged max projections of confocal image z-stacks are shown from representative spheroid pairs 7 hrs and 15 hrs after they were assembled. Scale bar: 50 μm. See S1 Fig for individual max projections plus DAPI staining.

To assess the spatial distribution of CMs and CFs, homotypic and heterotypic spheroid pairs were fluorescently stained for α-SA and Vim 7 and 15 hrs after assembly (**Fig 6, S1 Fig**). As expected, CM spheroids were α-SA-positive throughout with only a few contaminating Vim-positive cells. The fraction of Vim-positive cells increased as the fraction of CFs was increased in CM:CF spheroids (9:1 vs. 1:1). They were mainly located interstitially between the CMs, and some were found in a very thin later along the outer rim. CF spheroids were predominantly Vim-positive with only a few contaminating CMs. While homotypic spheroids appeared to coalesce evenly at 7 and 15 hrs, in heterotypic CM-CF pairs, distinct regions with α-SA or Vim predominance were present at both time points. Importantly, Vim-positive cells were noted within the α-SA-positive region (i.e., the CM spheroid), suggesting that some CFs migrate into CM spheroids over time. In contrast, CMs do not appear to migrate into CF spheroids, since α-SA-positive cells did not increase in the Vim-positive region over time.

### Action potential propagation in elongated microtissues

**Fig 7:**
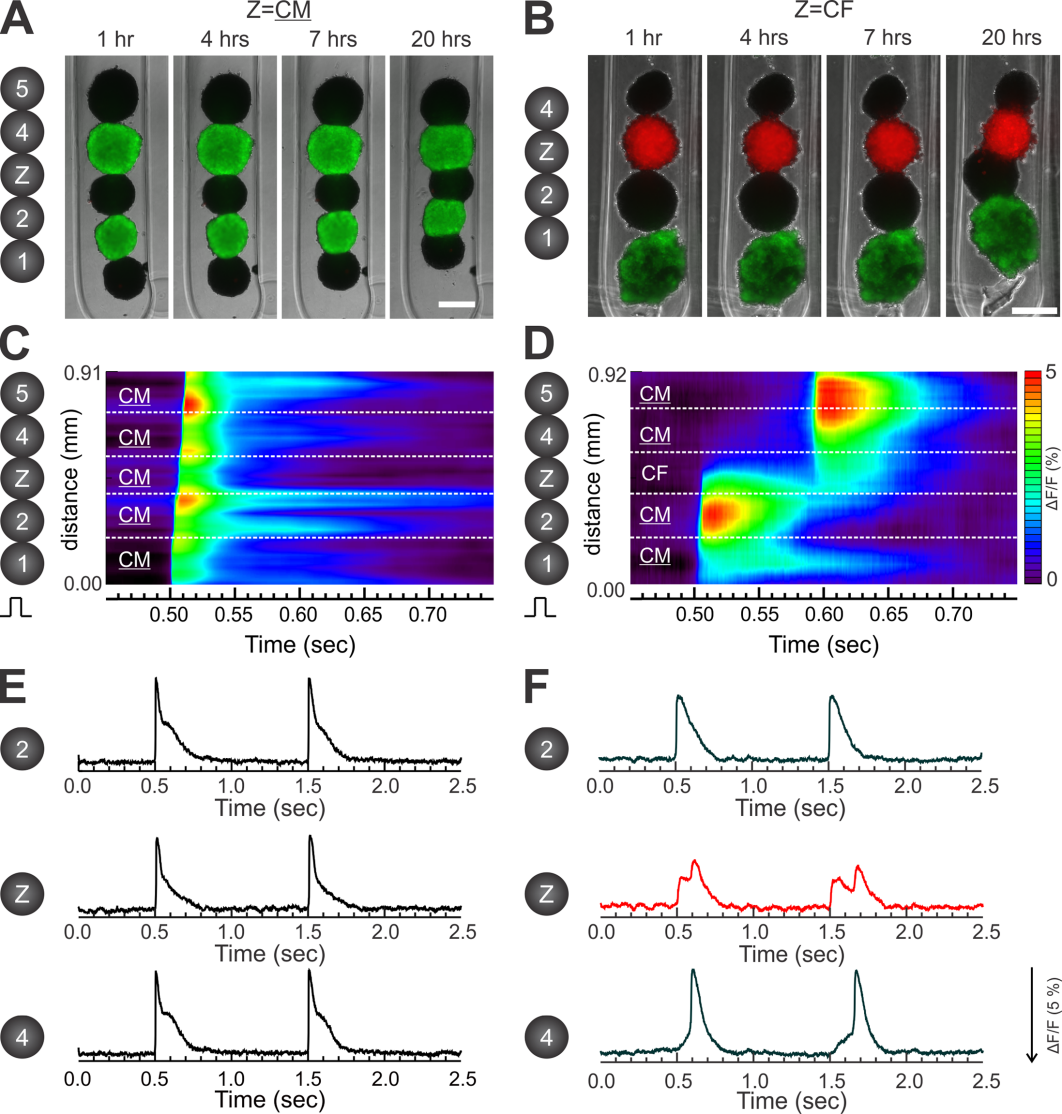
Electrotonic coupling and action potential propagation in elongated CM-CM-Z-CM-CM microtissues with a CM or a CF spheroid in the center. CM and CF spheroid building blocks were assembled in hydrogel troughs to generate elongated microtissues comprised of 2 CM spheroids on each end (labeled 1, 2, 4, 5) separated by either another CM spheroid (panels A, C, and E) or by a CF spheroid (panels B, D, and F) in the center (labeled as Z). CM denotes the inclusion of 5% CFs in the CM spheroids. **(A/B)** Representative merged phase contrast and immunofluorescent images that were acquired 1–20 hrs after assembly of the indicated spheroids (shown in a schematic to the left). Scale bars: 200 μm. The corresponding movie for the fusing microtissue shown in panel B is provided as S3 Movie. To better visualize the two configurations and spheroid fusion over time, some CMs were infected with Ad-GFP (green) and the CFs with Ad-RFP (red) prior to generation of the individual spheroids. For all subsequent optical mapping experiments, non-infected CMs and CFs were used. **(C/D)** Space-time plots of AP propagation acquired from indicated locations of elongated microtissues 7 hrs after assembly of indicated spheroids. The Y axis indicates the pixel locations and the X axis indicates time. The color bar represents depolarization of membrane potential (ΔF/F). The elongated tissue was stimulated from the bottom with a microelectrode. **(E/F)** The corresponding AP traces from panels C and D. Note that the activation pattern across the center CF spheroid (red trace) shows discontinuous conduction with two consecutive depolarizations in the AP trace: the first one is in synchrony with the AP recorded from the bottom CM (#2), while the second one is in synchrony with the AP from the top CM (#4).

**Fig 8:**
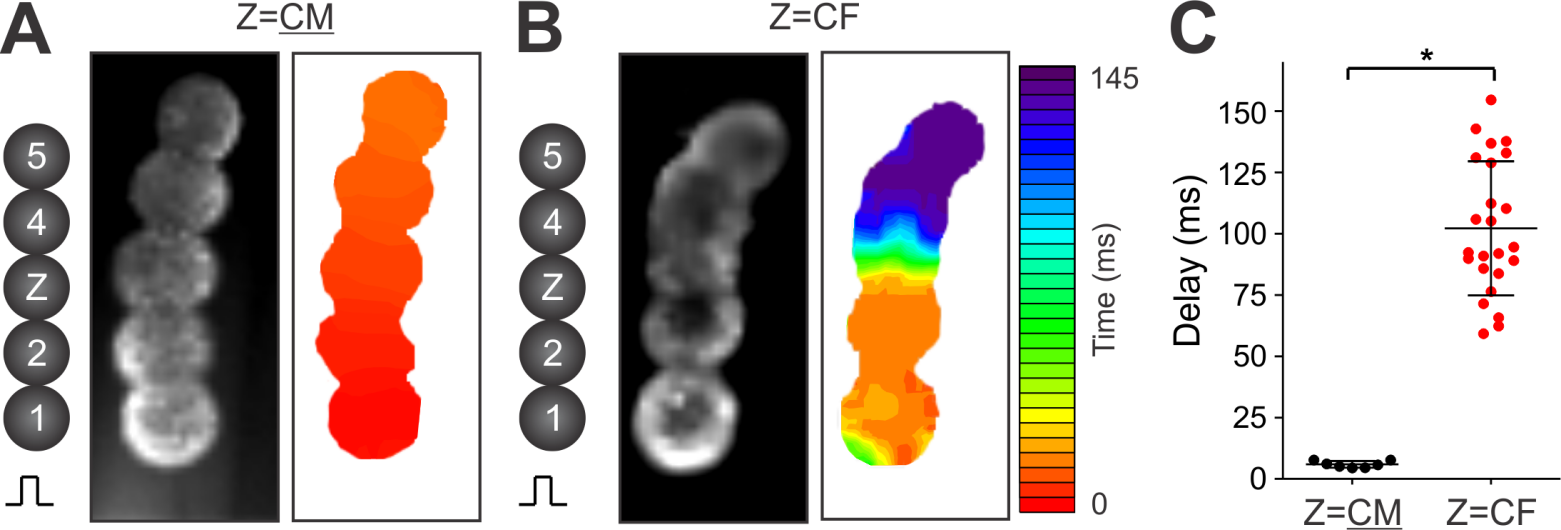
Action potential propagation maps and conduction delay in elongated CM-CM-Z-CM-CM microtissues with a CF or a CM spheroid in the center. CM and CF spheroid building blocks were assembled in hydrogel troughs as described for Fig 7. **(A/B)** Fluorescent images (*left*) and AP propagation maps (*right*) from elongated microtissues assembled from indicated spheroids. The corresponding movies are provided in S4 Movie. The color bar shown to the right of panel B represents time (in ms) for the AP propagation maps in both panels. **(C)** Time delay across a CM center spheroid (n = 7) and a CF center spheroid (n = 24). Mean±SD and individual data points. * P<0.05.

To determine whether the observed fusion of homotypic and heterotypic spheroids leads to functional coupling, we assessed AP propagation between spheroids using an optical mapping approach. To avoid a potential disturbance on AP propagation by electrical current flow from the stimulation electrode, we generated elongated microtissues comprised of multiple spheroids by fusing 2 CM spheroids on each end (labeled 1, 2, 4, 5) with another CM spheroid or a CF spheroid in the center (Z). In this configuration, the stimulation electrode evokes APs in spheroid #1, which then propagate into spheroid #2 and the rest of the microtissue ensuring the middle spheroid (Z) is sufficiently far away from the stimulation electrode. **Fig 7A and 7B** show images of the resulting microtissues in each configuration 1 to 20 hrs after assembly of 4 or 5 spheroids, some of which were CellTracker-labeled for enhanced visualization (see also **S3 Movie**). After 7 hrs of fusion, CM-CM-Z-CM-CM microtissues were transferred to the optical mapping apparatus. For each configuration representative space-time plots of V_m_ showing AP propagation (**Fig 7C and 7D**), corresponding AP traces recorded from the center three spheroids (**Fig 7E and 7F**), and AP propagation maps (**Fig 8A and 8B**) are shown.

In microtissues with Z = CM (**Figs 7C and 7E and 8A**), APs propagated across the entire tissue within 20 ms, indicating that electrotonic coupling is present after 7 hrs of fusion. In most of the microtissues with Z = CF (n = 26/35), APs propagated through the center CF spheroid but with a significant conduction delay (**Figs 7D and 7F and 8B)**. The space-time plot of V_m_ (**Fig 7D**) and AP traces (**Fig 7F**) show that AP propagation was not smooth but discontinuous with a step delay localized in the CF spheroid (Z). In the cases of propagation failure (n = 9/35), conduction block occurred within the center CF spheroid, as shown by a representative example in **S2 Fig**. The V_m_ trace from the CF spheroid exhibited two distinct depolarizations: the first one was in synchrony with the initial AP from CM spheroid #2 closer to the stimulation electrode (**Fig 7F**). Its amplitude gradually decreased toward the center and on the other side of the CF spheroid, indicating decremental conduction (**S3 Fig**). The second depolarization was in synchrony with the AP from CM spheroid #4 (**Fig 7F**), and its amplitude decreased as well indicating decremental conduction from spheroid #4 (**S3 Fig**). AP activation maps (**Fig 8A and 8B**) and time delay quantitation (**Fig 8C**) show markedly increased conduction delay across CF spheroids compared to CM spheroids (102.2 ± 27.4 ms vs 5.9±1.4 ms, P<0.05, see also **S4 Movie**).

### Computer simulations of action potential propagation

**Fig 9:**
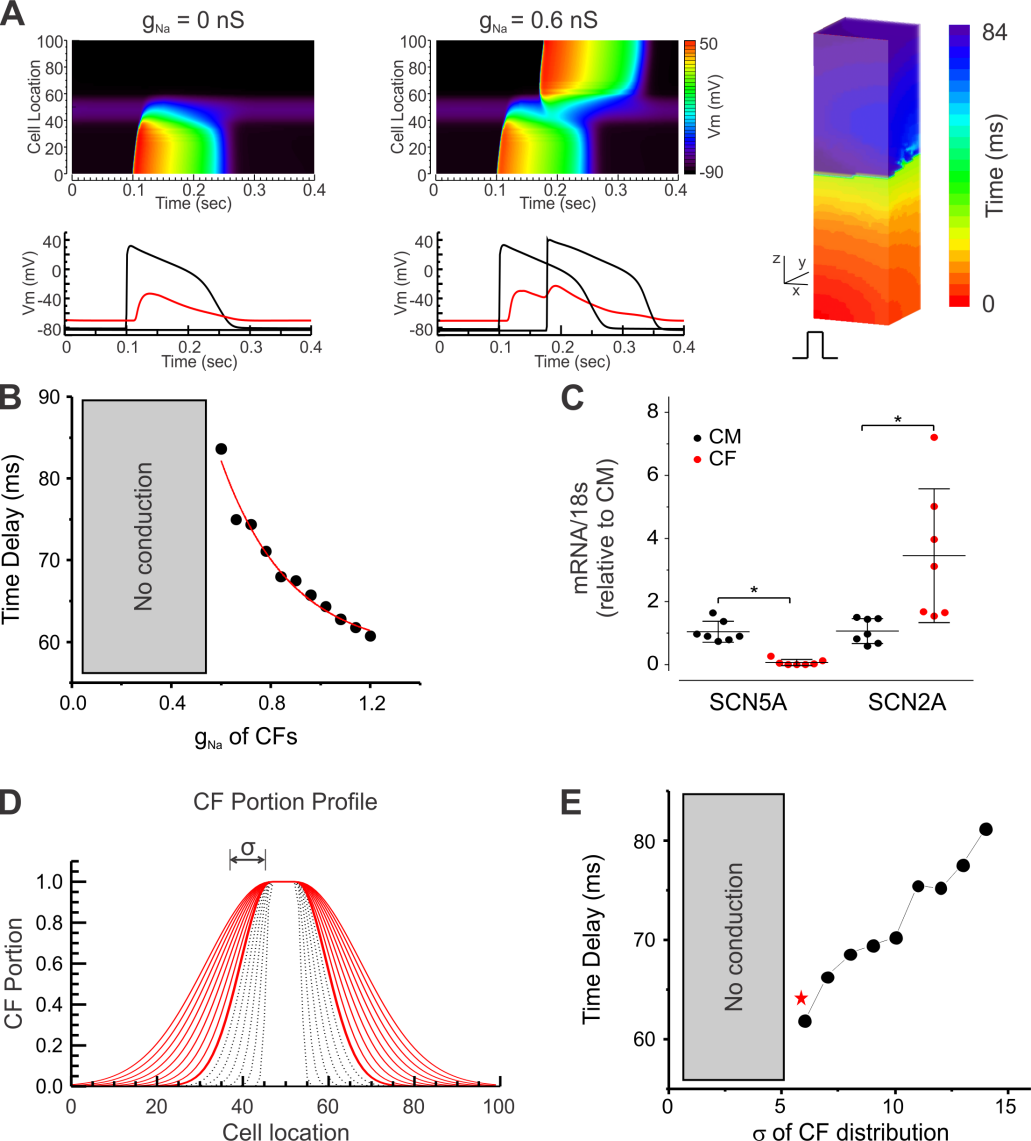
Computer modeling of action potential propagation across CF spheroids. **(A)** Sodium channel conductance (g_Na_) in CFs facilitates AP conduction: Conduction failure at the CF cube when CF g_Na_ = 0 but conduction across CF cubes when CF g_Na_ = 0.6 nS (see also S5 Movie). **(B)** Conduction time delay shortens with increasing g_Na_ in CFs. **(C)** Relative mRNA expression of TTX-sensitive (SCN2A) and TTX-resistant (SCN5A) Na^+^ channel α subunits assessed by qPCR in CF spheroids and expressed relative to CM spheroids. Mean±SD plus individual data points (n = 7 samples each). (**D/E)** Role of the spatial pattern of CM-CF boundary in AP conduction. CF portion profiles were generated using Gaussian function. (**D**). The CF density profiles of non-conduction cases are drawn with dotted lines and conduction cases are drawn with red lines. Note that the spread of the CM-CF boundary caused by infiltration of CFs paradoxically promotes conduction across CFs. The first conduction across CFs occur when σ = 6.0 (red star in **E**).

CF spheroids in our experimental condition demonstrated unique double depolarizations of decremental conduction with a step conduction delay. To better understand the mechanisms underlying these distinct conduction features across CFs, computer simulations were performed using a 3D box structure to represent the elongated tissue composed of 5 spheroids (see *Materials and methods* and **Fig 2**). Several CF characteristics can potentially modulate AP propagation across CF spheroids, such as ionic currents, coupling, and their spatial distribution. Previous studies reported that ventricular CFs express voltage-gated Na^+^ currents that are TTX-resistant and -sensitive [46, 47, 52]. Both types of Na^+^ currents were added to the CF model to investigate the potential roles of Na^+^ currents in facilitating conduction across CFs. When the conductance of Na^+^ currents (g_Na_) was set to zero, no AP could conduct across the CF region (**Fig 9A** left panels). However, when g_Na_ reached 0.6 (about 5% of CM g_Na_), the APs could propagate across the CF spheroids (**Fig 9A** right panels, see also **S5 Movie**). The AP in the CF region also exhibited distinct double depolarizations of membrane potential (red trace). The conduction delay was shortened with increased g_Na_ (**Fig 9B)**, indicating that Na^+^ channel expression in CFs plays important roles in conduction across CF spheroids. We confirmed mRNA expression of α subunits encoding both types of Na^+^ channels in our CF spheroids (**Fig 9C**). As expected, SCN5A encoding TTX-resistant Nav1.5 was expressed predominantly in CM spheroids based on the central role of Nav1.5 in CM excitation and impulse conduction. In contrast, SCN2A encoding TTX-sensitive Nav1.2 was 3.2-fold more abundant in CF spheroids than in CM spheroids.

Another important factor influencing AP propagation across CF spheroids is the spatial distribution of CFs at the CM-CF boundaries. To investigate this, we distributed CFs into the CM region of the 3D box structure using a Gaussian decay function (**Fig 9D**), representing migration of CFs into CM spheroids (**Fig 2A and 2B**). The effect of CF migration and spatial distribution on conduction was investigated by varying the width (σ) of the Gaussian function. Conduction was impaired when the CM-CF boundary was steep (gray dotted lines in **Fig 9D**) but successful when CF profile was wider (red lines), suggesting that increased CF migration into CM spheroids potentiates conduction. Note that conduction was potentiated by CF migration into the CM region, yet the conduction delay was increased (**Fig 9E**). Further increasing the width of CF distribution in the boundary (σ > 26) caused conduction failure (data not shown), suggesting a bimodal role of CF distribution in AP propagation. Our simulation results emphasize two important factors on conduction: 1) CF ionic Na^+^ currents and 2) spatial distribution of CM-CF in the boundary.

## Discussion

Multicellular spheroids are increasingly used as building blocks to bioengineer larger, more complex tissues with cellular heterogeneity, organization, and other biomimetic intricacies of natural tissue. In this study, we developed elongated cardiac microtissues by fusing individual spheroids comprised of CMs and/or CFs. These building blocks were assembled in defined configurations to first characterize the fusion of multicellular cardiac aggregates with variable cellular composition and then examine the effect of CF spatial distribution on cardiac AP propagation. We show that coculture of CFs with CMs (at different ratios) changes spheroid size and the kinetics of spheroid fusion, and that the fusion of heterotypic pairs (a CM and a CF spheroid) is significantly accelerated compared to fusion of their homotypic counterparts, with some CF migration into the CM spheroids but not vice versa. Spheroids fused into larger microtissues showed an electrical syncytium with synchronized APs within 7 hrs of culture. In this engineered platform, we provide evidence for electrotonic coupling and AP propagation across CF blocks, albeit with a substantial time delay compared to propagation across CM blocks. The observed propagation could be computationally modeled, and our simulations point to important roles of the spatial pattern of the CM-CF boundary and CF Na^+^ channels in facilitating and modulating AP conduction across CF regions.

### Directed fusion of cardiac spheroids into larger heterocellular microtissues

Microtissue spheroids can undergo directed fusion into macrotissues of desired shapes, and the kinetics and extent of fusion are important to biofabrication [23, 24]. Characterization of spheroid fusion has mainly been carried out in homotypic pairs or groups of monocultured spheroids [50, 51, 53–57] and cocultured spheroids [58, 59] and has included human chondrocytes, MCF-7, adipose derived stem cells, olfactory ensheathing cells, human pluripotent stem cells, and cocultures of smooth muscle cells and endothelial cells. Some studies have also looked at the fusion of spheroids with intact tissue such as the myocardium [60, 61]. Comparison of the kinetics of homotypic pairs to each other and/or heterotypic fusions have not been studied extensively [57, 62, 63], and the dynamics of tissues comprised of different cardiac cell types have not been characterized. In this study, we compared the two major cardiac cell types and then combined them in coculture to better mimic the complex morphology and heterotypic cell-cell interactions that naturally occur in the myocardium. Regulation of CM function by CFs is known to occur *in vivo* [43, 64]. Here, we show that CFs also appear to influence CMs in accelerating the fusion kinetics of cardiac spheroids: homotypic CF pairs fused faster than homotypic CM pairs, but interestingly, coculture of CMs with just 10% CFs significantly increased the rate of CM fusion (**Figs 4 and 5**). The most striking finding from the examination of cardiac spheroid pair fusion was the rapid kinetics of fusion between heterotypic CM-CF spheroid pairs (**Fig 5**). CMs and CFs are known to be intermingled in the heart, with virtually every CM bordering one or more CFs [31]. Our results suggest that self-assembled CMs and CFs may favor a cohesive and intermixed morphology. Homotypic CM:CF(1:1) pairs also fused more quickly than their monocultured counterparts (**Fig 5**), pointing toward the preference of cohesion among CMs and CFs in 3D cocultures.

Tissue spheroids undergoing fusion are thought to merge and coalesce analogous to liquid droplets [25], and there is qualitative and quantitative support for tissue liquidity in both the developmental context of the heart and in *in vitro* assays of two fusing spheroids [65]. Tissue fusion is believed to follow the differential adhesion hypothesis (DAH, [66]), according to which cells in non-adherent cultures aggregate into spheroids to minimize surface tension and the cell type expressing the strongest intercellular adhesions in cocultured spheroids form the center with less adhesive cell types forming an outer layer. Multiple spheroids have been shown to fuse over time to a more energetically favorable configuration of one larger spheroid, and therefore spheroid fusion has also been thought to follow the DAH [63, 67]. In our study, CMs express much more N- and pan-cadherins than CFs (**Fig 3F**), which have been implicated in enhancing cohesion[67]. Contrary to what the DAH would predict, CMs did not form an inner core surrounded by CFs. Only very few Vim-positive CFs were observed at the outer rim in our self-assembled cocultured spheroids and spheroids pairs, while most CFs resided interstitially between the CMs (**Fig 6**, **S1 Fig**). This is consistent with previous reports from us [13] and others [68] and points to additional mechanisms playing critical roles in cell sorting of CMs and CFs. More recent work has suggested that preformed spheroids do not necessarily follow the DAH and that tissue fusion kinetics can be affected by tissue maturation [58, 62]. Preformed spheroids have a more mature cytoskeleton, increased ECM [69, 70], and interplay amongst cell-cell, cell-matrix, and cytoskeletal interactions can affect fusion. It has also been shown that longer culture slows the kinetics of spheroid fusion [51]. Interestingly, CMs cocultured with CFs are more spherical and more tightly packed (**Fig 3**) and fuse more rapidly than in monoculture (**Fig 5**). This observation seems to parallel effects seen with actin modulators: human mesenchymal stem cell spheroids treated with cytochalasin D and Y-27632 showed disintegrated outer actomyosin spheroid boundaries and slowed fusion [51]. This may point to a potential role of cytoskeletal maturation and architecture in CM:CF coculture spheroids that requires further investigation.

While we focused in this study on the self-sorting properties of CMs and CFs, which are not necessarily dependent on relative cell motility, motility is likely an important factor in spheroid fusion. Compared to fibroblasts, cardiomyocytes are much less motile in 2D culture (e.g., [71]). Fibroblast motility is known to be inversely related to cell density in monolayer cultures which has been attributed to contact inhibition [72]. It is also reduced in cellular 3D aggregates but still present [73]. Our observation of Vim-positive cells within the CM spheroid of heterotypic CM-CF spheroid pairs (**Fig 6**) suggest CF migration, consistent with early observations made in heterotypic pairs of spherical cell aggregates comprised of chick embryonic ventricular fibroblasts and myocytes [73].

The guided fusion approach for cardiac spheroidal building blocks developed in this study is relevant for biofabrication of larger tissue constructs with controlled configurations of multiple cell types, mimicking the complex morphology and physiological tasks of natural tissues. Continuous advances in other technologies will enhance its utility, broaden implementation and increase throughput. For example, magnetic approaches could be used to manufacture versatile types of spheroids with distinct cellular spatial distribution and to spatially guide spheroid assembly and fusion [74–76]. A limitation of our current approach in generating larger tissues is the manual process of moving and assembling the spheroids. In that regard, pick and place instruments can aid in the assembly of scaffold-free building parts into larger multicellular microtissues of diverse shapes [77], and 3D spheroid bioprinting approaches are developed for the fabrication of cardiac tissue patches [78], vascular grafts [79] and other cardiovascular applications with increased throughput [80].

The insights gained in this study using a newly developed cardiac *in vitro* model based on guided spheroid fusion will be useful for the development of complex heterocellular engineered cardiac tissue constructs and their engraftment via tissue fusion, and they have implications for arrhythmogenesis in cardiac disease and repair (see below). This spheroid-based approach offers flexibility in experimental design and the ability to test translationally relevant parameters altered in the diseased heart (such as cellular composition, ratios, distribution and activation states) [44]. However, it is important to note that scaffold-free spheroid-based cardiac microtissues lack anisotropic tissue structure, tension, and some of the biomechanical properties that are important for engineered heart tissues intended for other purposes (such *in vivo* implantation) because they do not include any natural or synthetic biomaterials that can support cellular attachment and alignment, transmit load, and provide stiffness [81].

### Electrical integration of 3D elongated microtissues constructed with spheroids as building blocks

The primary objective of engineered tissue platforms is to mimic the natural organ system. We show that CM spheroids can fuse within 7 hrs of 3D culture into larger tissues with an electrical syncytium, one of the most important features of heart tissue because it is required for rapid and synchronized contraction to maximize blood circulation. Our data show that impulses can propagate across fused CM spheroids with minimal delay between the junctions of spheroids (**Figs 7C and 7E** and **8A**, **S4 Movie**), pointing to the potential of cardiac tissues to be used in biofabrication to create larger cardiac tissue constructs [78] that can be implanted for *in vivo* cardiac repair [9].

CF spheroids can also electrically couple with CM spheroids within 7 hrs of directed fusion, as evidenced by impulse propagation through CF spheroids albeit with substantial delay (**Figs 7D and 7F** and **8B and 8C, S4 Movie**). Previous studies in the intact heart [38, 82, 83] and 2D cell culture [84] reported that CFs can support AP propagation. A time delay of ~ 60 ms across a 302 μm wide CF region (up to 6–8 CFs) was reported in micropatterened 2D culture [84]. In our 3D model, the time delay was substantially longer (~110 ms, **Fig 8C**) across 255±38 μm diameter CF spheroids (derived from **Fig 3B**). Based on the internuclear distance (**Fig 3D**), our CF blocks had 22±5 CFs across their diameter. Cells can contact and form electrical coupling in all directions in 3D culture, so that APs can still propagate around poorly coupled areas in 3D. This zigzag propagation in 3D may potentiate AP propagation but may increase conduction delay. Alternatively, electrophysiological characteristics of CFs may be different in the 3D environment compared to 2D, where CFs tend to differentiate into myofibroblasts, especially on stiff cell culture substrates [85]. Further characterization of CFs in 3D are needed.

### Implications to proarrhythmogenic roles of cardiac fibroblasts

CFs are important mediators of structural and functional remodeling in cardiac disease that have been closely linked to cardiac arrhythmias especially post-MI [41, 86–88]. Traditionally, infarct areas are considered electrical insulators or obstacles causing conduction block and reentry formation. However, growing evidence from *in vitro* studies [84, 89–91], *in vivo* studies [83, 92], and computer simulations [93–97] suggest that CFs can electrically couple with CMs and with each other. Coupling between CF and CM has been suggested to slow down AP conduction by causing rapid dissipation of electrical currents from CM to CFs [98]. Our data showing AP conduction across CFs, although with substantial delay (**Figs 7**and **8, S4 Movie**), are consistent with the notion that CFs can form electrical coupling with CM and CF and alter AP conduction. Slow conduction has been long implicated to facilitate reentry and ventricular arrhythmias particularly in the border zone of MI [99–101]. Slow conduction in MI is thought to be caused by reduced gap junctions and Na^+^ currents of CMs, and disruption of CM alignment by fibrosis. Our findings suggest an alternative explanation of slow conduction caused by compact volume of CFs, which can heighten the risk for reentry formation. Importantly, the spatial distribution of CFs in the boundary (border zone) also influences conduction. The conventional view is that dispersed fibroblasts in the myocardium (such as in the border zone of myocardial infarction) slow down conduction through zig-zag propagation and increase risks for conduction block. However, CF migration into CM regions in our engineered microtissues (**Fig 6**) created interspersed spatial distributions of CFs, which slowed down conduction even more yet paradoxically supported conduction in our computer simulations (**Fig 9D and 9E**). This phenomenon of potentiation of conduction by diffuse CF distribution can be explained by the source-sink relationship of depolarizing currents: when CMs in the boundary are well-coupled with many neighboring CMs, the electrotonic current flow from the center CFs to the boundary CMs dissipates quickly to the neighboring CMs, which reduces the likelihood to depolarize V_m_ above the threshold needed for an AP to be generated in the CM region. Upon interference of coupling between neighboring CMs by CFs in the boundary, the current flow from the center CFs could sufficiently elevate V_m_ of CMs to initiate APs. This paradoxical improvement of conduction has also been shown experimentally in cultured monolayers of CMs with a gap junction blocker that reduces dissipation of depolarizing currents to the neighboring CMs thereby potentiating action potential propagation [102].

In addition to the spatial distribution of CFs in the border, ionic currents of non-excitable cells coupled to CMs have been suggested to modulate AP generation, shape, and conduction, as suggested by *in vitro* studies in micropatterned 2D culture and computer simulations [103, 104]. The computer simulation studies by Jousset et al [94] showed AP propagation across a compact CF region in 2D simulation with steady-state currents in CF that had larger depolarizing currents and a higher resting membrane potential than MacCannell’s CF model used in the present study, which can facilitate AP conduction through a compact CF region. Previous studies by multiple groups reported that CFs express Na^+^ channels [46, 47, 105]. Our findings suggest that Na^+^ channel expression in CFs may support conduction by providing additional depolarizing currents that maintain V_m_ of CFs at higher voltage and inject sufficient currents to the neighboring CMs to fire APs. We confirmed the presence of both TTX-sensitive (Nav1.2) and -resistant Na^+^ channels (Nav1.5) in CFs (**Fig 9C**). Our simulations support a critical role of Na^+^ channel conductance in facilitating AP propagation across CFs (**Fig 9A and 9B**, **S5 Movie**). The different kinetics of TTX-resistant Na^+^ currents having faster activation/inactivation and lower V_1/2_ than TTX-sensitive Na^+^ currents [106], may contribute to AP conduction in CFs. However, the results of the present computer simulation should be interpreted with caution. It does not include patch clamp studies of *I*_*Na*_ and does not investigate potential roles of the two types of Na^+^ channels or specific isoforms in computer simulation. We also recognize the limitation of the simplified simulation scheme of the elongated tissue, and a more realistic finite element method will be required to study AP propagation across complex scars in myocardial infarction, which takes into account the heterogeneous conduction and interfusion of the cells. However, the realistic infarct model simulation is beyond the scope of this manuscript. Further studies are needed to understand the influence of different Na^+^ and other ionic currents on the membrane potential of CFs, which may modulate impulse propagation and conduction delay. The impact of age and the activation state of CFs also requires further investigation in light of their emerging [68, 107] and more established [44, 91, 108] roles in the functional regulation of CMs, respectively.

## Conclusions

We developed cardiac 3D elongated microtissues by fusing individual 3D spheroids comprised of CMs and/or CFs. The fusion of heterotypic pairs consisting of a CM and a CF spheroid was accelerated compared to fusion of their homotypic counterparts and associated with migration of CFs into the CM spheroids. These spheroids fused into longer elongated microtissues that have an electrical syncytium within 7 hrs of culture. In this engineered platform, we provide evidence for electrotonic coupling and AP propagation via CFs but with large conduction delay compared to CMs. The observed propagation could be computationally modeled, and our simulations indicate that the spatial pattern of the CM-CF boundary and CF ionic currents, particularly Na^+^ channels, are important modulators of AP conduction across CF volumes.

## Supporting information

S1 Fig **CM and CF distribution in homotypic and heterotypic spheroid pairs 7 hrs (A) and 15 hrs (B) after fusion.** Cryosections of homotypic (X-X) and heterotypic (X-Y) pairs of spheroids of the indicated cellular compositions were fluorescently double-stained with antibodies recognizing α-sarcomeric actinin (α-SA) and vimentin (Vim) to visualize CMs (green) and CFs (red), respectively. Nuclei were stained with DAPI (blue). Max projections of confocal image z-stacks are shown (individually and merged) from representative spheroid pairs 7 hrs (A) and 15 hrs (B) after the spheroid pairs were assembled. Scale bars: 50 μm. Merged images for α-SA and Vim are shown in Fig 6.
(TIF)

S2 Fig Example of an elongated CM-CM-CF-CM-CM microtissue with failed action potential propagation across the CF center despite electrotonic coupling with CMs. Space-time plots of AP propagation (**A**) and corresponding AP traces (**B**) acquired from indicated locations of an elongated microtissue with a CF spheroid in the center (Z). In this representative example (n = 9/35), the amplitude of V_m_ depolarization gradually decreased through the CF spheroid and failed to initiate APs on the opposite side. Note that a small depolarization is still visible from the CM spheroids on the opposite side (#4, bottom trace), indicating that this CM spheroid is still electrically coupled with the CF spheroid. S4 Movie further illustrates failed AP propagation (*right panel*).
(TIF)

S3 Fig Overlapped V_m_ traces from elongated CM-CM-CF-CM-CM microtissues illustrating decremental AP conduction. **(A)** Experimental AP traces acquired by optical mapping illustrate representative examples of microtissues with conduction (*left*) and with conduction failure (*right*) across a CF center spheroid. The red traces indicate recordings from the center of the CF spheroid. The amplitude of the first AP upstroke decrease gradually, indicating decremental conduction within the CF spheroid (*left*). The gradual decrease of upstroke amplitudes is also seen in microtissue with conduction failure (*right*). **(B)** Computer simulation results of conduction (g_Na_ = 0.6, *left*) and conduction failure (g_Na_ = 0.1, *right*) that both also replicate the decremental conduction feature seen experimentally.
(TIF)

S1 Movie Contraction movie of 3D elongated microtissue. The movie was acquired from elongated microtissues 7 hrs after assembly of 5 CM spheroids. The elongated microtissue was stained with the voltage sensitive dye di-4 ANEPPS and stimulated at 1 Hz. The movie shows the initial bright flashes of action potential followed by contraction of the microtissue.
(MP4)

S2 Movie Time course of directed self-assembly of a heterotypic CM-CF spheroid pair. After 3 days in 3D culture as individual spheroids, a spheroid comprised of CMs and a spheroid comprised of CFs that had been infected in suspension with an adenovirus encoding RFP 2 hrs prior to initial cell seeding in 3D culture, were replated in pairs and allowed to fuse. Merged phase contrast and immunofluorescent time lapse images were acquired every hour over 19 hrs post assembly. Scale bar: 100 μm.
(AVI)

S3 Movie Time course of the fusion of an elongated microtissue generate from individual CM and CF spheroids. After 3 days in 3D culture as individual spheroids, four spheroids comprised of either CMs (CM, non-fluorescent), CMs expressing GFP (CM_GFP_, green fluorescent) or CFs expressing RFP (CF_RFP_, red fluorescent) were replated in trough-shaped hydrogel recesses in the following configuration: CM_GFP_-CM-CF_RFP_-CM. To generate the indicated individual spheroids, prior to cell seeding in 3D culture, a subset of CMs and CFs were incubated in suspension for 2 hrs with adenoviruses encoding GFP and RFP, respectively. Both infected and uninfected CM spheroids also contained 5% of uninfected CFs that were added during initial cell seeding to match the conditions used for the optical mapping experiments. Merged phase contrast and immunofluorescent time lapse images were acquired every hour over 19 hrs post assembly in the trough. Scale bar: 200 μm.
(AVI)

S4 Movie Experimental movie files illustrating action potential propagation in elongated CM-CM-Z-CM-CM microtissues. The movies were acquired from elongated microtissues 7 hrs after assembly of 4 CM spheroids and either another CM spheroid in the center (Z = CM) or a CF spheroid in the center (Z = CF). The color bar represents depolarization of membrane potential (ΔF/F). The elongated tissue was stimulated from the bottom with a microelectrode. Note that AP conduction was very rapid across the CM spheroid (*left*). In most microtissues with a CF spheroid in the center, conduction was also observed but with a pronounced delay (*middle*). An example of a microtissue with conduction failure across the CF center is shown to the *right*. See Figs 7C–7F and 8A–8C for detailed maps and conduction delay.
(MP4)

S5 Movie 3D box computational movie file illustrating the importance of sodium channel conductance in CFs for AP propagation across multiple CFs. Three representative examples of elongated tissue simulations from a 3D box model with either CMs in the center (labeled”CM only”) or CFs in the center (labeled “CF Middle”), for which the Na^+^ channel conductance (g_Na_) was set at g_Na_ = 0.6 nS (*left*) or g_Na_ = 0 nS (*right*). See Fig 9A and 9B for detailed maps and conduction delay.
(MP4)

S1 Appendix Computer simulation code. Numerical calculations were performed with a custom-developed software using Interactive Data Language (IDL) programing environment (Harris Geospatial Solutions) and CUDA toolkit (Nvidia). The core cellular ionic current routines and electrotonic current flow between cells were written in ansi-C with CUDA for multi-core-GPU calculation. Visualization routines were written in IDL. The compressed file includes ansi-C, CUDA, wrapper routines for IDL, and Readme.txt of instruction for building and using this software package.
(ZIP)

S2 Appendix Time course of changes in intersphere angle and length of homotypic and heterotypic spheroid pairs. After 3 days in 3D culture as individual spheroids, CM, CM:CF (at 9:1 and 1:1 ratios), and CF microtissues were replated in pairs and allowed to fuse. Representative phase contrast images were acquired over 19 hrs post assembly of homotypic (X-X) pairs, in which X are either CM, CM:CF(9:1), CM:CF(1:1), or CF spheroids, and of heterotypic (X-Y) pairs comprised of a CM and a CF spheroid. For each of the indicated spheroid pairs, intersphere angle and long axis length were measured, and normalized pair length was calculated (relative to length 1 hr after replating). The three parameters are shown in separate worksheets. Graphic representations of these results are shown in Fig 5.
(XLSX)
