# Supplementary figures and images for: Directed fusion of cardiac spheroids into larger heterocellular microtissues enables investigation of cardiac action potential propagation via cardiac fibroblasts
Source: PLoS One. 2018 May 1;13(5):e0196714. doi: 10.1371/journal.pone.0196714 (PMC5929561; doi:10.1371/journal.pone.0196714)

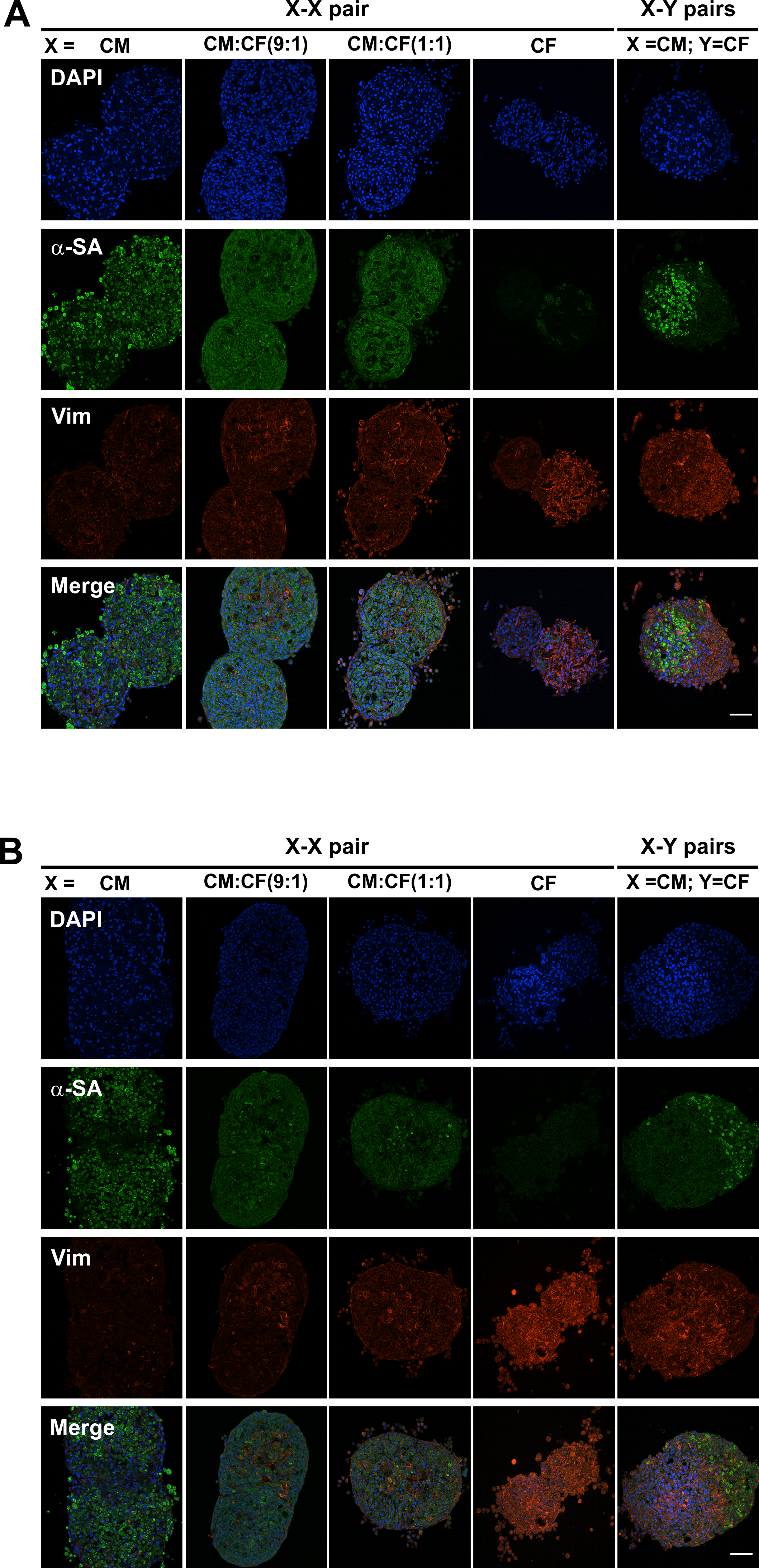

Supplement: S1 Fig — CM and CF distribution in homotypic and heterotypic spheroid pairs 7 hrs (A) and 15 hrs (B) after fusion. Cryosections of homotypic (X-X) and heterotypic (X-Y) pairs of spheroids of the indicated cellular compositions were fluorescently double-stained with antibodies recognizing α-sarcomeric actinin (α-SA) and vimentin (Vim) to visualize CMs (green) and CFs (red), respectively. Nuclei were stained with DAPI (blue). Max projections of confocal image z-stacks are shown (individually and merged) from representative spheroid pairs 7 hrs (A) and 15 hrs (B) after the spheroid pairs were assembled. Scale bars: 50 μm. Merged images for α-SA and Vim are shown in Fig 6. (TIF) [file pone.0196714.s001.tif]

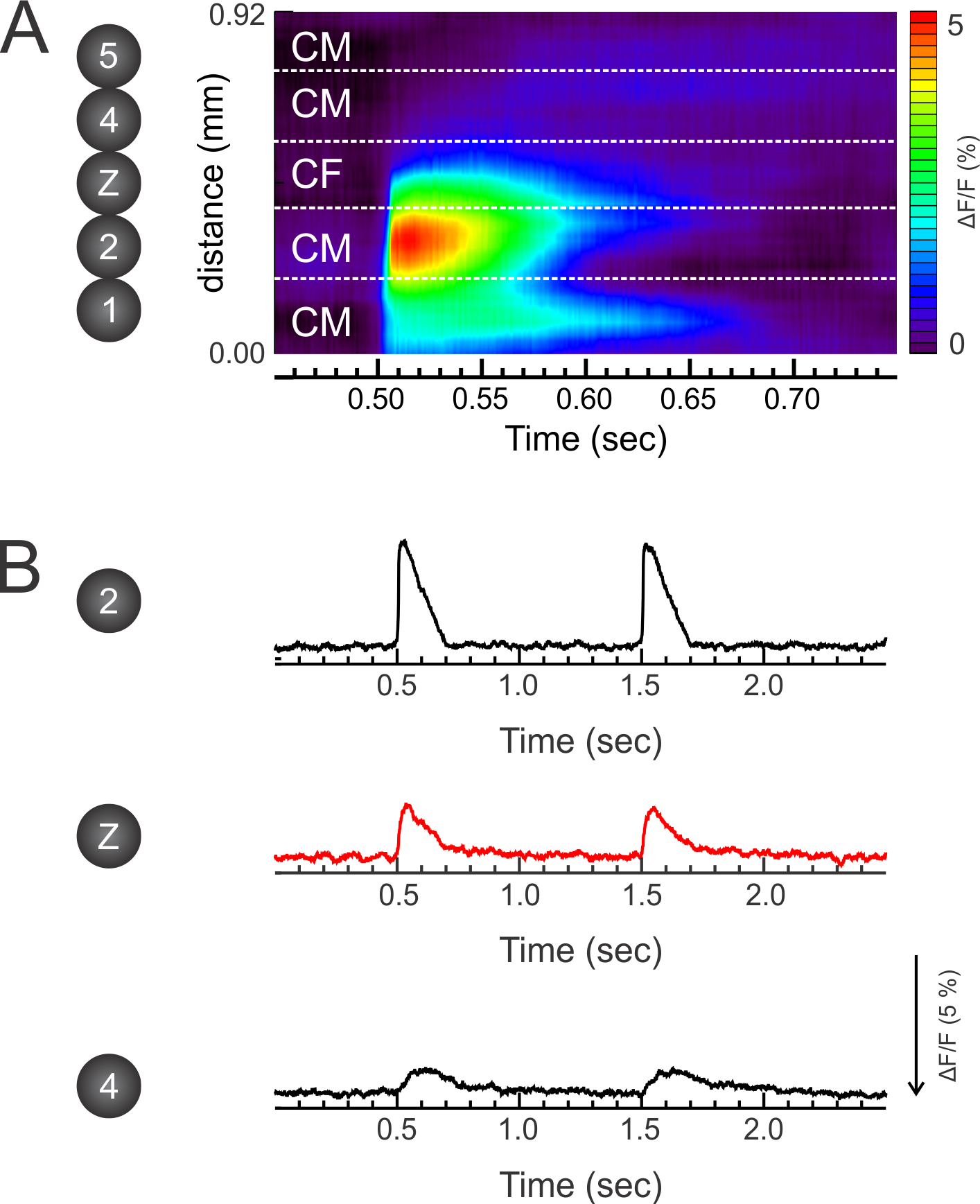

Supplement: S2 Fig — Space-time plots of AP propagation (A) and corresponding AP traces (B) acquired from indicated locations of an elongated microtissue with a CF spheroid in the center (Z). In this representative example (n = 9/35), the amplitude of Vm depolarization gradually decreased through the CF spheroid and failed to initiate APs on the opposite side. Note that a small depolarization is still visible from the CM spheroids on the opposite side (#4, bottom trace), indicating that this CM spheroid is still electrically coupled with the CF spheroid. S4 Movie further illustrates failed AP propagation (right panel). (TIF) [file pone.0196714.s002.tif]

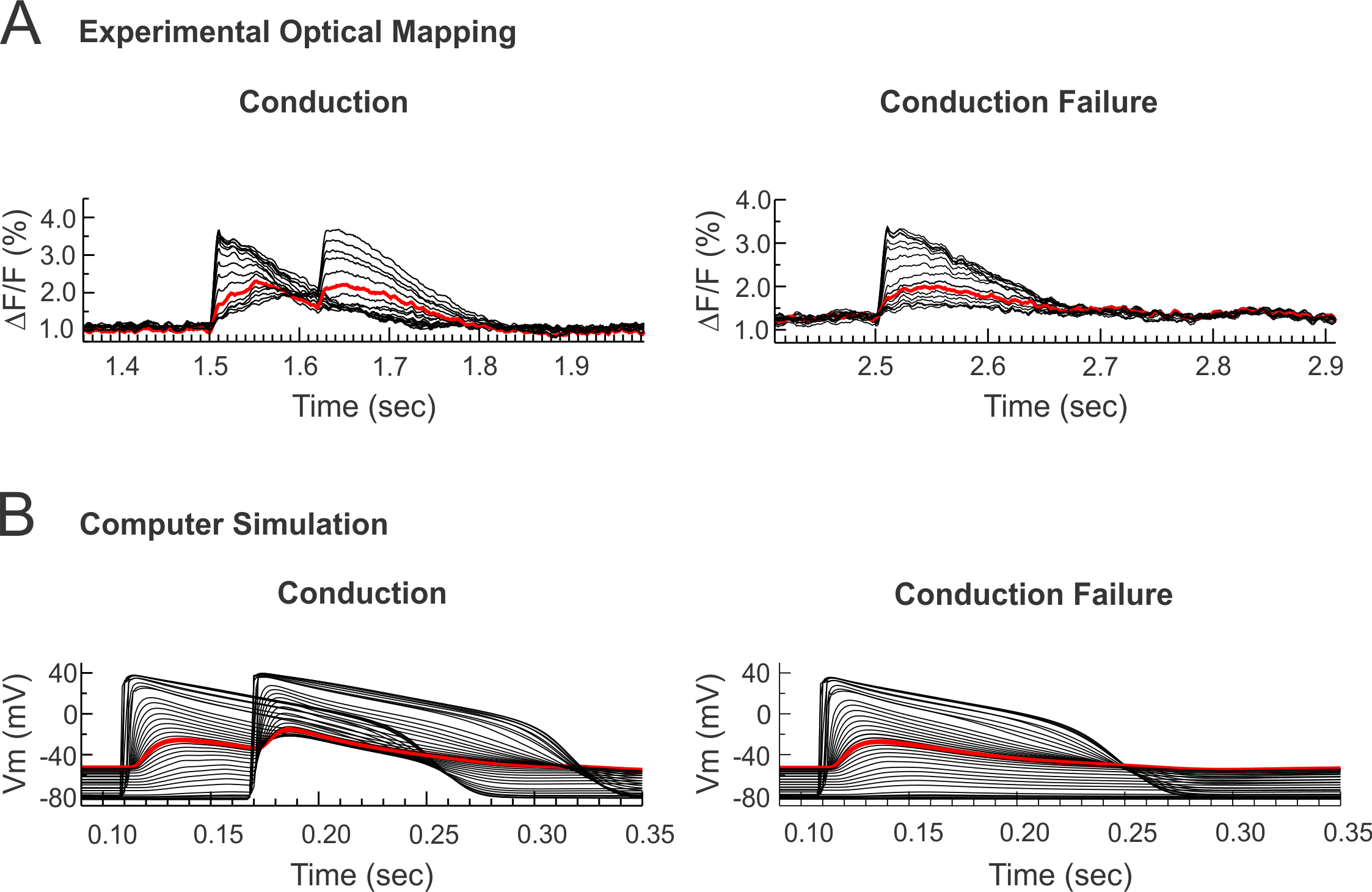

Supplement: S3 Fig — (A) Experimental AP traces acquired by optical mapping illustrate representative examples of microtissues with conduction (left) and with conduction failure (right) across a CF center spheroid. The red traces indicate recordings from the center of the CF spheroid. The amplitude of the first AP upstroke decrease gradually, indicating decremental conduction within the CF spheroid (left). The gradual decrease of upstroke amplitudes is also seen in microtissue with conduction failure (right). (B) Computer simulation results of conduction (gNa = 0.6, left) and conduction failure (gNa = 0.1, right) that both also replicate the decremental conduction feature seen experimentally. (TIF) [file pone.0196714.s003.tif]
